# Vemurafenib-resistance via de novo RBM genes mutations and chromosome 5 aberrations is overcome by combined therapy with palbociclib in thyroid carcinoma with BRAF^V600E^

**DOI:** 10.18632/oncotarget.21262

**Published:** 2017-09-24

**Authors:** Zeus A. Antonello, Nancy Hsu, Manoj Bhasin, Giovanni Roti, Mukta Joshi, Paul Van Hummelen, Emily Ye, Agnes S. Lo, S. Ananth Karumanchi, Christine R. Bryke, Carmelo Nucera

**Affiliations:** ^1^ Laboratory of human thyroid cancers preclinical and translational research, Division of Experimental Pathology, Cancer Research Institute, Cancer Center, Department of Pathology, Beth Israel Deaconess Medical Center, Harvard Medical School, Boston, MA, USA; ^2^ Department of Pathology, Center for Vascular Biology Research, Beth Israel Deaconess Medical Center, Harvard Medical School, Boston, MA, USA; ^3^ Cytogenetics Laboratory, Department of Pathology, Beth Israel Deaconess Medical Center, Harvard Medical School, Boston, MA, USA; ^4^ Bioinformatic and Systems Biology Unit, Department of Medicine, Beth Israel Deaconess Medical Center, Harvard Medical School, Boston, MA, USA; ^5^ Department of Medicine and Surgery, University of Parma, Parma, Italy; ^6^ Center for Cancer Genome Discovery, Dana Farber Cancer Institute, Harvard Medical School, Boston, MA, USA; ^7^ Department of Medicine, Beth Israel Deaconess Medical Center, Harvard Medical School, Boston, MA, USA

**Keywords:** papillary thyroid cancer preclinical model, BRAFV600E, chromosome 5, combined therapy with vemurafenib and palbociclib, drug resistance

## Abstract

**Purpose:**

Papillary thyroid carcinoma (PTC) is the most frequent endocrine tumor. BRAF^V600E^ represents the PTC hallmark and is targeted with selective inhibitors (e.g. vemurafenib). Although there have been promising results in clinical trials using these inhibitors, most patients develop resistance and progress. Tumor clonal diversity is proposed as one mechanism underlying drug resistance. Here we have investigated mechanisms of primary and secondary resistance to vemurafenib in BRAF^WT/V600E^–positive PTC patient-derived cells with P16^-/-^ (CDKN2A^-/-^).

**Experimental Design:**

Following treatment with vemurafenib, we expanded a sub-population of cells with primary resistance and characterized them genetically and cytogenetically. We have used exome sequencing, metaphase chromosome analysis, FISH and oligonucleotide SNP-microarray assays to assess clonal evolution of vemurafenib-resistant cells. Furthermore, we have validated our findings by networks and pathways analyses using PTC clinical samples.

**Results:**

Vemurafenib-resistant cells grow similarly to naïve cells but are refractory to apoptosis upon treatment with vemurafenib, and accumulate in G2-M phase. We find that vemurafenib-resistant cells show amplification of chromosome 5 and *de novo* mutations in the RBM (RNA-binding motifs) genes family (i.e. RBMX, RBM10). RBMX knockdown in naïve-cells contributes to tetraploidization, including expansion of clones with chromosome 5 aberrations (e.g. isochromosome 5p). RBMX elicits gene regulatory networks with chromosome 5q cancer-associated genes and pathways for G2-M and DNA damage-response checkpoint regulation in BRAF^WT/V600E^-PTC. Importantly, combined therapy with vemurafenib plus palbociclib (inhibitor of CDK4/6, mimicking P16 functions) synergistically induces stronger apoptosis than single agents in resistant-cells and in anaplastic thyroid tumor cells harboring the heterozygous BRAF^WT/V600E^ mutation.

**Conclusions:**

Critically, our findings suggest for the first time that targeting BRAF^WT/V600E^ and CDK4/6 represents a novel therapeutic strategy to treat vemurafenib-resistant or vemurafenib-naïve radioiodine-refractory BRAF^WT/V600E^-PTC. This combined therapy could prevent selection and expansion of aggressive PTC cell sub-clones with intrinsic resistance, targeting tumor cells either with primary or secondary resistance to BRAF^V600E^ inhibitor.

## INTRODUCTION

The BRAF^V600E^ mutation is the most frequent genetic alteration in papillary thyroid carcinoma (PTC) (∼60%) and in melanoma (∼60-70%) [1, 2]. BRAF^V600E^ is a prognostic biomarker of recurrence, tumor aggressiveness and mortality in patients with melanoma, PTC and hairy cell leukemia [3-10]. It is implicated in the tumor aggressiveness of anaplastic/undifferentiated thyroid cancer (ATC) [5], a devastating disease that is still untreatable. Although highly selective inhibitors of BRAF^V600E^, including the first FDA-approved orally available vemurafenib [11] have been tested in clinical trials for BRAF^V600E^–positive cancers with promising results [2], tumor primary resistance and development of secondary resistance to BRAF^V600E^ inhibitors in patients that were initially responding have been reported [12]. Different compensatory mechanisms have been shown to promote this resistance bypassing pharmacologic inhibition of BRAF^V600E^ via the triggering of intracellular signaling cascade pathways which lead to reactivation of phospho(p)-ERK1/2. Some of these include: elicitation of aberrant autocrine loops through the overexpression of HER3 receptor [13], over-activation of PI3K/AKT and c-Met pathways [14], or JAK/STAT3 signaling [15]. Other mechanisms are also linked to eIF4F complex formation which is associated with reactivation of ERK1/2 signaling [16] or to persistent activation of p-mTOR and p-S6 ribosomal proteins signaling [17]. Resistance to BRAF^V600E^ inhibitors is also mediated by dimerization of aberrantly spliced BRAF^V600E^ that dimerize in a RAS-independent manner [18]. NRAS mutations, BRAF^V600E^ amplifications, MEK1/2 mutations, and overexpression of genes including COT, PDGFRB, and others, play a role in the resistance to BRAF^V600E^ inhibitors [19]. Also, we have recently found that copy number gain of MCL1 (chromosome 1q) in metastatic BRAF^V600E^-PTC cells are associated with resistance to vemurafenib treatment [20]. Moreover, we showed that the loss of P16 gene (also named P16^INK4^ or CDKN2A, chromosome 9p21) in PTC samples might be an important event for metastatic potential [20]. P16 is a crucial negative regulator of cell cycle by inhibiting the assembly of CDK4/6 complexes and Rb phosphorylation during G1 phase [21]. Because of P16 loss in invasive thyroid tumor cells, targeting P16 downstream cell cycle effectors using CDK4/6 selective inhibitors in combination with BRAF^V600E^ inhibitor was rationalized. We decided to use palbociclib, a selective inhibitor of CDK4/6 FDA-approved for treatment of patients with advanced breast cancers [22, 23]. Overall, because of the occurrence of tumor resistance, new therapeutic options are urgently needed against cancers which are resistant to BRAF^V600E^ inhibitors, including BRAF^V600E^-positive metastatic PTC. The mechanistic link between vemurafenib treatment and the development of resistance in PTC is still not well understood. Cancer is an evolutionary system and based on cytogenetically diverse clones [24]. Tumor heterogeneity might be fundamental for tumor capacity to expand resistant clones driven by different selective pressures including anti-cancer therapies [25]. Understanding thyroid carcinoma cytogenetic heterogeneity and genomic alterations emerging during therapy with BRAF^V600E^ inhibitors is crucial to improving clinical studies and will help investigate mechanisms of tumor progression. We selected vemurafenib-resistant BRAF^WT/V600E^-positive and P16^-/-^ PTC patient-derived cells, assessed their cytogenetic and genomic profiles, and validated the therapeutic efficacy of combined treatment with vemurafenib plus palbociclib to efficiently target vemurafenib-resistant cells. Our study proposes a novel strategy to treat BRAF^V600E^-positive invasive PTC resistant to BRAF^V600E^ inhibitors.

## RESULTS

### Model of vemurafenib resistance in PTC patient-derived cells harboring the heterozygous BRAF^V600E^ mutation and with biallelic deletion of P16 (P16^-/-^)

**Figure 1:**
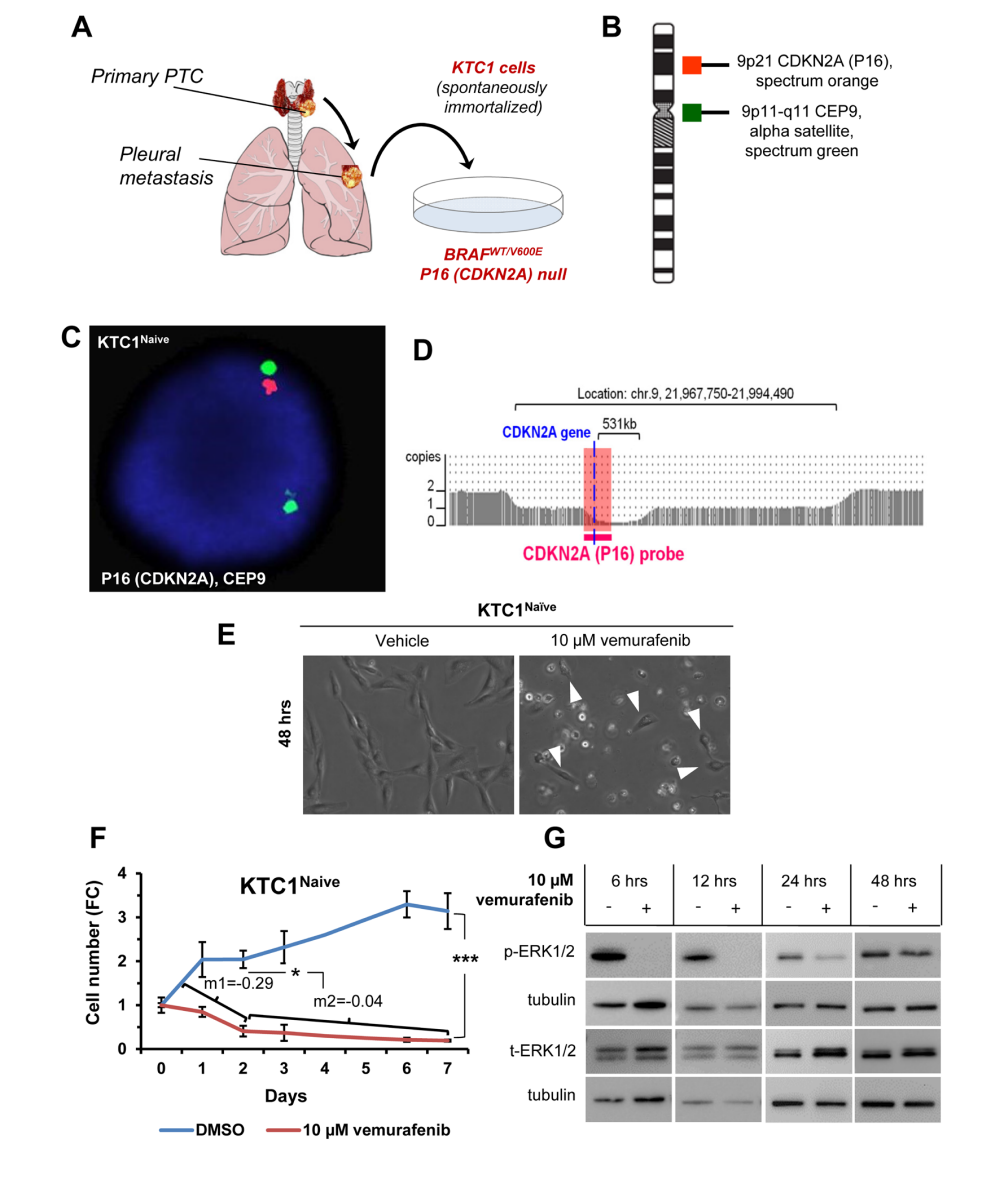
Model of primary resistance to vemurafenib using PTC patient-derived cells harboring the heterozygous BRAF^V600E^ mutation and with P16 (CDKN2A) deletion **A.** KTC1 cells are spontaneously immortalized cell derived from the pleural effusion of a BRAF^V600E^ positive recurrent papillary thyroid carcinoma (PTC). **B.** Probe design for the detection of P16 (CDKN2A) by fluorescence *in situ* hybridization (FISH) in KTC1 cells. **C.** FISH analysis for the detection of P16 (CDKN2A) gene in KTC1 cells. **D.** Microarray analysis of KTC1 cells (pink). Zoom in view of the CDKN2A gene region of chromosome 9 showing the biallelic deletion of 9p21. The larger 3.0 Mb deletion on one chromosome 9 takes out the CDKN2A gene and the entire segment covered by the orange FISH probe, while the smaller 531 kb deletion also results in deletion of CDKN2A but leaves intact a small portion of the region covered by the FISH probe. This explains why a single small red CDKN2A signal was detected by FISH. All above results were validated by two independent replicate measurements. **E.** Phase contrast images of KTC1 cells treated with 10 µM vemurafenib or DMSO (vehicle) for 48 hours (hrs) show sub-population of cells resistant to treatment (arrowheads). These results were validated at least by three independent replicate measurements. **F.** Growth curve based on KTC1 cell count shown as fold change (FC) in the presence of 10 µM vemurafenib or vehicle (DMSO). Angular coefficient (m) values between 0 and 2 days (m1); between 2 and 7 days (m2) are shown: cell death rate was significantly reduced by 6.8-folds beyond 2 days by vemurafenib treatment. These data represent the average ± standard deviation (error bars) of four independent replicate measurements (*p < 0.05, **p < 0.01, ***p < 0.001). **G.** Representative western blot analysis of KTC1 cells treated with 10 µM vemurafenib at the indicated time points shows that phospho(p)-ERK1/2 protein expression levels are not reduced in surviving cells compared to vehicle-treated cells. These results were validated at least by three independent replicate measurements.

Here, we have developed a new model (Figure 1A) to investigate primary and secondary resistance to vemurafenib using the human spontaneously immortalized KTC1 cells derived from the pleural effusion from a recurrent and RAI refractory PTC patient. Uniquely, KTC1 cells are the only PTC patient-derived cells, to the best of our knowledge, which harbor with heterozygous status (as occurs in PTC samples with BRAF^V600E^) the constitutively active mutant kinase BRAF^V600E^ [20]. Therefore, these cells represent a helpful preclinical model for therapeutics validations and for investigating mechanisms of resistance to BRAF^V600E^ inhibitors for human aggressive/refractory PTC. Additionally, these cells show biallelic deletion of P16 (chromosome 9p) (Figure 1B-1D, Supplementary Table 1) and TP53^WT^. They also have copy number neutral loss of heterozygosity on chromosome 2p, 2q and 7q (Supplementary Table 1) and gain of chromosome 5p (which includes hTERT –human telomerase reverse transcriptase- gene) and 17p (Supplementary Table 1). Our findings indicated that vemurafenib blocked orthotopic KTC1 tumor growth in an early intervention model but tumor size was not reduced over time suggesting primary tumor resistance [20]. Here we found that most naïve cells died upon treatment within 48-96 hours (Figure 1E). However, the surviving cells showed a significantly reduced cell death rate on longer time points (Figure 1F, angular coefficients ‘m1’ at 0-2 days versus ‘m2’ at 2-7 days upon vemurafenib treatment) and rebound in phospho(p)ERK1/2 protein levels (Figure 1G), indicating that the few surviving cells could have intrinsic (primary) resistance. As the antitumor activity of BRAF^V600E^ inhibitors requires near-complete inhibition of ERK1/2 activation, minimal reactivation of ERK1/2 signaling might lead to tumor progression. Reactivation of ERK1/2 phosphorylation has been shown to occur steadily upon vemurafenib treatment in human cancer cells [13, 26]. We have previously showed dose-response curves (IC50, 50% maximal inhibitory concentration) for vemurafenib in different human thyroid cancer cells [20]. Here, in a time course of anti-BRAF^V600E^ therapy using KTC1 cells, we have assessed pERK1/2 protein levels as readout of BRAF^V600E^ kinase activity inhibition, and we found that vemurafenib has an acute effect in the early hours of treatment but pERK1/2 is progressively less inhibited in the following time points compared vehicle-treated control cells (Figure 1G). Additionally, we analyzed pERK1/2 protein expression levels in other 4 human thyroid carcinoma-derived cell lines, PTC or ATC-derived cells, with either heterozygous or homozygous mutation for BRAF^V600E^ or with BRAF^WT/WT^ (Supplementary Figure 1). Interestingly, we observed in heterozygous BRAF^V600E^ cell lines (KTC1 and SW1736) increased pERK1/2 protein levels from 6 to 12 hours independently of their histologic origin of the tumor cell line. Whereas the human thyroid carcinoma cells with only BRAF^WT^ (i.e. TPC1) (with no BRAF^V600E^) (Supplementary Figure 1) showed an increase in pERK1/2 protein expression levels upon vemurafenib treatment when compared to vehicle treatment; this result is similar to melanoma cell lines with BRAF^WT^ which showed paradoxical phenomenon of ERK/12 phosphorylation activation by BRAF^V600E^ inhibitors [27].

### Vemurafenib treatment selects BRAF^V600E^-positive and P16^-/-^ PTC patient-derived cells clones with unchanged growth rate

**Figure 2:**
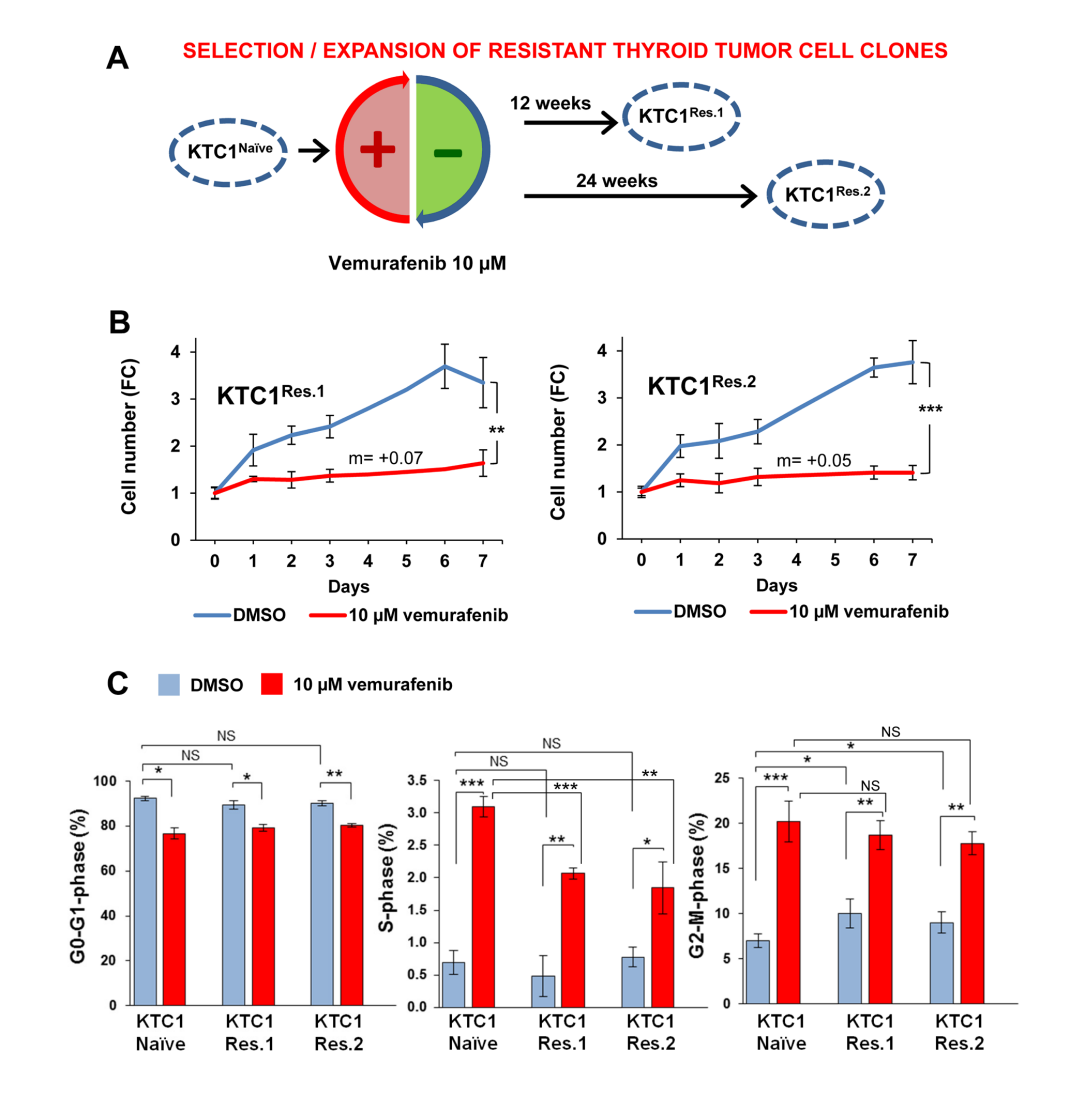
Clonal selection and expansion of PTC patient-derived cells with BRAF^V600E^ and P16^-/-^ in the presence of vemurafenib treatment **A.** Experimental model of clonal expansion of KTC1 cells with primary resistance to vemurafenib: two independent batches of resistant cells, KTC1^Res.1^ and KTC1^Res.2^, were exposed to 10 µM vemurafenib for 12 or 24 weeks, respectively, to select vemurafenib-resistant clones. **B.** Growth curve of KTC1^Res.1^ and KTC1^Res.2^ cells treated with 10 µM vemurafenib or vehicle (DMSO). Vehicle-treated KTC1^Res.1^ and KTC1^Res.2^ cells show similar growth rate compared to KTC1^Naive^ cells (Figure 1F). Vemurafenib-treated resistant cells grew significantly slower compared to vehicle-treated control cells. Vemurafenib-treated resistant cells grew with a constant rate (slope/gradient analysis, m value=+0.07 or =+0.05, respectively) compared to KTC1^Naive^ cells (negative m values reported in Figure 1F). These data represent the average ± standard deviation (error bars) of four independent replicate measurements (*p < 0.05, **p < 0.01, ***p < 0.001). **C.** Percentage of cells in G0-G1, S and G2-M phases in KTC1^Naive^, KTC1^Res.1^, and KTC1^Res.2^ when exposed to 10 µM vemurafenib or vehicle (DMSO) for 48 hrs: vemurafenib treatment induced a significant increase of either naïve or resistant cells in S and G2-M phase compared to vehicle-treated cells. Vehicle-treated resistant cells significantly increased in G2-M phase compared to vehicle-treated naïve cells (KTC1^naïve^=6.33±0.78; KTC1^Res1^=9.53±1.61, *p*-value=0.023; KTC1^Res2^= 8.65±1.15, *p*-value=0.032). These data represent the average ± standard deviation (error bars) of two independent replicate measurements (*p < 0.05, **p < 0.01, ***p < 0.001, NS=not significant).

In order to investigate the mechanisms of primary resistance to vemurafenib treatment and understand their relationship with the potential occurrence of secondary resistance, we have expanded the subpopulation of KTC1 cells capable to survive to acute therapeutic doses of vemurafenib (Figure 2A). We have selected two independent vemurafenib-resistant tumor cells batches by applying cycles of high doses of vemurafenib alternated by expansion of the surviving cells (Figure 2A). Most KTC1 cells died upon treatment with vemurafenib within 48-96 hours however the few surviving cells (Figure 1E, arrows), when biochemically assayed for pERK1/2 levels showed no difference between vehicle and vemurafenib treatment (Figure 1G), indicating that they have primary resistance to vemurafenib. To expand and analyze this cell subpopulation with intrinsic primary resistance, KTC1 cells were exposed to vemurafenib, and then the few surviving cells were expanded without treatment (Figure 2A) in order to avoid bias toward the selection of secondary mutations which might specifically trigger cell cycle progression. When we analyzed vemurafenib-resistant KTC1 cells for growth following a week-long vemurafenib-sustained treatment, we found that these cells showed a net increased number over the time but with a significantly slower growth rate compared to vehicle-treated cells (best fitting curves equations: y = 0.0722x + 1.0444 and y = 0.0513x + 1.0576) (Figure 2B). Instead, vemurafenib-naïve cells (Figure 1F) showed a reduction of the total cell number as shown by the negative growth rate (see Figure 1F, best fitting curve days 0-2: y = -0.2959x + 1.3438 and days 2-7: y = -0.0457x + 0.449). In addition, when resistant cells (Figure 2B) were exposed to the vehicle, they did not show any significant change in growth rate compared to vehicle-treated naïve KTC1 cells (Figure 1F). Analysis of cell cycle by BrdU (5-bromo-2-deoxyuridine)/PI (propidium iodide) combined assay showed that both resistant cells batches exposed to vehicle have a significantly higher cell percentage distributed in G2-M phase compared to naïve cells (Figure 2C, 1.51 and 1.36 fold-change, respectively). In addition to these results, we found that cyclin B1, a crucial regulator for the transition of cells from G2 to M phase, showed substantial increased mRNA expression levels (1.2 fold-changes) in the vemurafenib-resistant KTC1 cells compared to the vemurafenib-naïve KTC1 cells when exposed to vehicle treatment.

### Oligonucleotide-SNP microarray, metaphase chromosome and FISH analyses of naïve and vemurafenib-resistant thyroid carcinoma cells show karyotypic heterogeneity and expansion of clones with chromosome 5 aberrations

**Figure 3:**
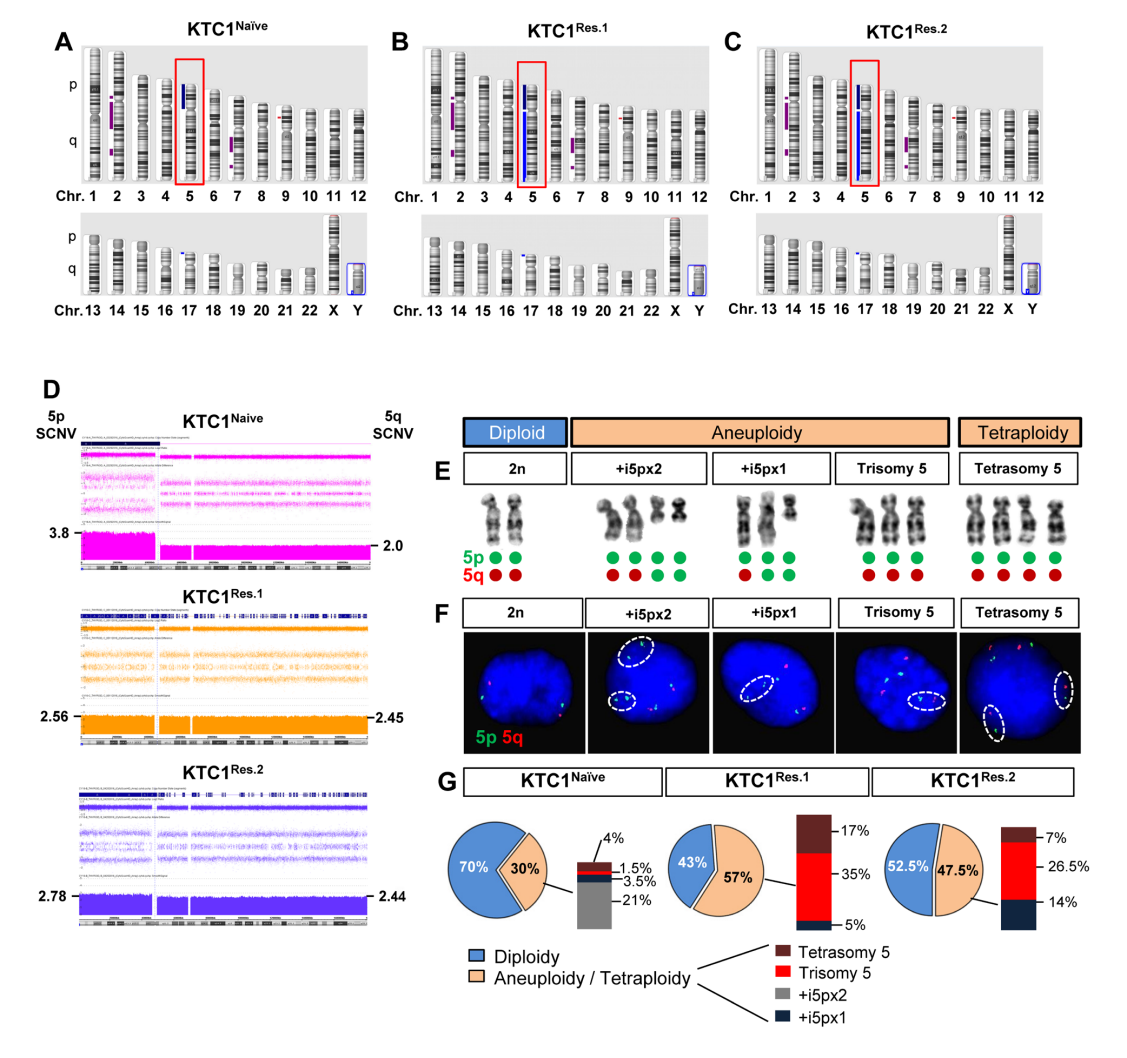
Vemurafenib-resistant PTC patient-derived cells with BRAF^V600E^ and P16^-/-^ show an increased tetraploidy/aneuploidy and expansion of clones with chromosome 5 amplification **A.**-**C.** Ideogram of an Affymetrix oligonucleotide-SNP microarray analysis of the chromosomes landscape of KTC1^Naive^, KTC1^Res.1^, and KTC1^Res.2^ cells showed copy number gain of chromosome 5 (red box) and on 17p, loss on chromosome 9p and copy number neutral loss of heterozygosity on chromosomes 2 and 7. KTC1^Res.1^ and KTC1^Res.2^ cells acquire copy number gain of 5q compared to KTC1^Naive^ cells. **D.** Affymetrix oligonucleotide-SNP microarray analysis with zoom in of somatic copy number variations (SCNV) of chromosome 5 in KTC1^Naive^ (pink), KTC1^Res.1^ (orange) and KTC1^Res.1^ (blue) cells. KTC1^Res.1^ (2.45 copies) and KTC1^Res.2^ (2.44) showed extra copies of chromosome 5q compared to the KTC1^Naive^ cells (2 copies). KTC1^Naive^ cells showed 3.8 extra copies of 5p (produced by two copies of one 5p isochromosome) in 45% of the cells in the sample. KTC1^Res.1^ cells showed 2.56 copies of chromosome 5p in 44% of the cells and 2 extra copies of 5p (produced by a 5p isochromosome) in 17% of the cells in the sample. KTC1^Res.2^ cells showed 2.78 copies of chromosome 5 in 45% of the cells and 2 extra copies of 5p (produced by a 5p isochromosome) in 5.5% of the cells in the sample. All these findings were validated by at least by two independent experiments with replicates measurements. **E.** Clones with respect to chromosome 5 identified in KTC1^Naive^, KTC1^Res.1^ and KTC1^Res.2^ cells. KTC1 cells showed karyotype with either diploidy, or aneuploidy or tetrasomy (due to tetraploidy) of chromosome 5. Green and red dots exemplify how these karyotypes are visualized by FISH in panel F. **F.** Chromosome 5 clones assessed by fluorescence *in situ* hybridization (FISH) analysis in KTC1^Naive^, KTC1^Res.1^ and KTC1^Res.2^ cells. Specific probes identify chromosome 5p (green) and 5q (red). **G.** Quantification of chromosome 5 clones detected by FISH in KTC1^Naive^, KTC1^Res.1^ and KTC1^Res.2^ cells.

In order to assess tumor cell heterogeneity and clonal evolution, we performed Affymetrix oligonucleotide-SNP microarray assay (Figure 3A-3D), metaphase chromosome analysis (Figure 3E), fluorescence in situ hybridization (FISH, Figure 3F), and exome sequencing (Supplementary Tables 2-4) in the 2 independent populations of resistant KTC1 cells (KTC1^Res.1^ and KTC1^Res.2^, Figure 2A) and compared to the parental vemurafenib-naive KTC1 cells (KTC1^Naive^). The oligonucleotide-SNP microarray assay identified copy number neutral loss of heterozygosity on chromosome 2p, 2q and 7q and gain of chromosome 5p (Figure 3A-3D) and 17p (Figure 3A-3C). These somatic copy number variations in vemurafenib-resistant KTC1 cells and naïve KTC1 cells were similar (including the biallelic deletion of P16) except for the gain of the entire chromosome 5 detected in the vemurafenib-resistant KTC1 cells but not in the naïve KTC1 cells (Figure 3D, Supplementary Table 1). Interestingly, no other chromosome acquired a copy number variation (gain or loss) in resistant cells. The duplication on chromosome 17p, as well as the 2p, 2q and 7q neutral loss, occurred prior to the verumafenib treatment in KTC1^Naïve^ cells and remained unchanged in the resistant cells. Furthermore, we found aneuploidy and tetrasomy (due to tetraploidy) of chromosome 5 (Figure 3E-3G, Supplementary Figure 2, Supplementary Table 1). We confirmed and quantified this result by analyzing two hundred interphase nuclei for cell condition by FISH (Figure 3F) with probes for chromosome 5, revealing that 70% and 30% of the KTC1^Naïve^ cells showed diploidy, or aneuploidy/tetraploidy, respectively. Specifically, KTC1^Naïve^ cells included diverse clones with trisomy of chromosome 5, with one or two copies of supernumerary *isochromosome of the short arm of chromosome 5 (+i5p),* or clones with tetrasomy due to tetraploidy (Figure 3F-3G, Supplementary Figure 2, Supplementary Table 1). Importantly, the presence of extra chromosomes has been reported to lead to genomic instability [28]. Remarkably, following the long-term sustained treatment with vemurafenib, resistant KTC1 cells reduced the diploid clones 1.3-1.7 fold-change and increased the aneuploid/tetraploid clones 1.5-1.9 fold-change compared to the naïve cells. In particular, KTC1^Res.1^ cells showed 43% and 57% diploid or aneuploidy/tetraploidy karyotype, respectively, and KTC1^Res.2^ cells showed 52.5% and 47.5% diploid or aneuploidy/tetraploidy karyotype, respectively (Figure 3F-3G).

### Vemurafenib-resistant BRAF^V600E^-PTC patient-derived cells acquire *de novo* mutations in RBM genes

**Figure 4:**
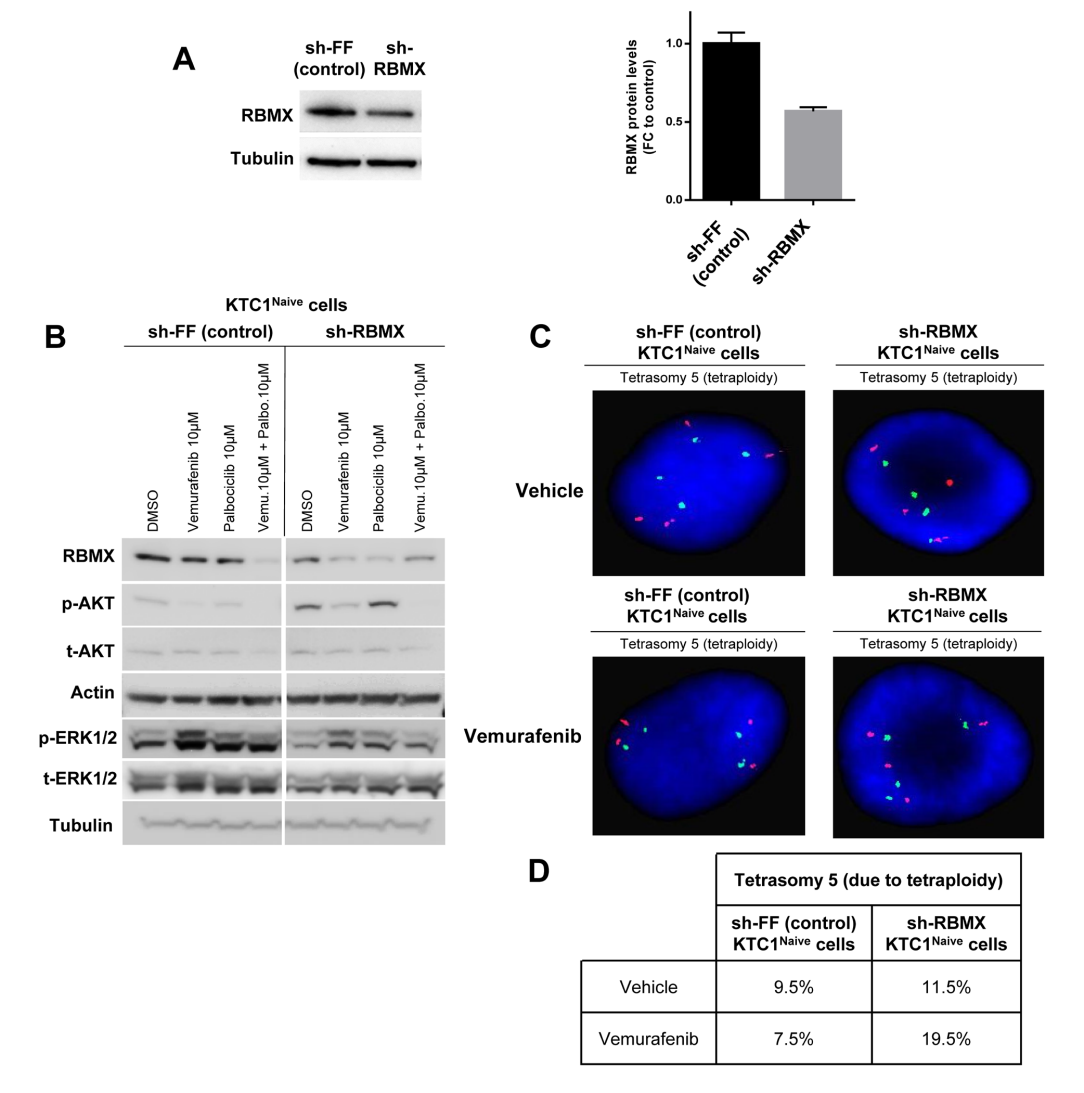
RBMX knock-down contributes to tetraploidy in PTC patient-derived cells with BRAF^V600E^ and P16^-/-^ treated with vemurafenib **A.** Representative western blotting analysis of RBMX knockdown (sh-RBMX) compared to control (sh-FF) and densitometry quantification (FC=fold change) of RBMX protein levels at 48 hrs post seeding in BRAF^V600E^-KTC1^Naive^ cells. **B.** Representative western blotting analysis of RBMX, pAKT, tAKT, pERK1/2 and tERK1/2 protein expression levels in KTC1^Naive^ cells with RBMX knockdown (sh-RBMX) compared to shRNA control (sh-FF) cells at 48 hrs. These results were validated by two independent replicate measurements. **C.** Fluorescence *in situ* hybridization (FISH) analysis of KTC1^Naive^ cells with RBMX knockdown compared to sh-control cells. **D.** Quantification of clones by interphase fluorescence *in situ* hybridization (FISH) analysis in KTC1^Naive^ cells engineered to express sh-RBMX or sh-control (shFF). Cells were treated for 48 hrs with either vehicle or 10 µM vemurafenib. These results were validated quantifying 200 cells by two independent replicate measurements.

To further investigate the mechanisms which contribute to the survival and expansion of vemurafenib-resistant KTC1 cells, we also analyzed by exome sequencing the occurrence of secondary and somatic copy number variations. Exome sequencing is able to detect single nucleotide changes and defines with precision the allelic fraction of the identified mutation. As internal control we verified the mutant BRAF^V600E^ allele fraction, and as expected we found that it resulted 50% (along with 50% of wild type BRAF allele) in both naïve and resistant KTC1 cells (Supplementary Table 2). More importantly, the exome sequencing analysis after appropriate filter application displayed mutations (by allelic fraction analysis) in the RBM genes only in the vemurafenib-resistant cells but not in the vemurafenib-naïve cells. In particular, we found an in-frame deletion in the RBM10 gene (chromosome X, 1537_1539delGAG, E513del) with 87% of allele fraction in the KTC1^Res.1^ cells (Supplementary Table 3) and a missense mutation in the RBMX gene (chromosome X, 499C>G, P167A) with 33.3% of allele fraction in the KTC1^Res.2^ cells (Supplementary Table 4). The RBMX^499C>G^ mutation shows protein change as follows: P167A (proline (P) to alanine (A)). Proline has major roles in the proper tertiary structure folding of proteins. Loss of a proline and substitution with the non-polar alanine may be predicted to result in potential loss of an important structural motif in the RBMX protein RBMX (also named hnRNP-G or RBMXP1). RBMX plays an important role to protect cells against DNA damage [28] and for chromosome segregation [29] and might contribute, therefore, to vemurafenib resistance. To test this hypothesis, we knocked-down wild-type (WT) RBMX using the most efficient shRNA (Figure 4A) among the two tested (data not shown) in the naïve KTC1 cells compared to the sh-control cells. Importantly, silencing of RBMX (sh-RBMX) showed an increase of p-AKT protein levels in naïve KTC1 cells compared to the sh-control cells when exposed to vehicle or single agent treatments (Figure 4B). FISH analysis demonstrated that RBMX knockdown resulted *per se* in a modest increase of tetraploidization (9.5% in sh-control cells vs. 11.5% in sh-RBMX cells) and in a substantial increase (2.6 fold-changes) in tetraploidy in the presence of vemurafenib treatment, from 7.5% in the sh-control cells to 19.5% in the sh-RBMX cells (Figure 4C-4D).

### RBM genes elicit regulatory networks with up-regulated genes in BRAF^V600E^-PTC compared to BRAF^WT^-PTC clinical samples which are involved in tumor survival

**Figure 5:**
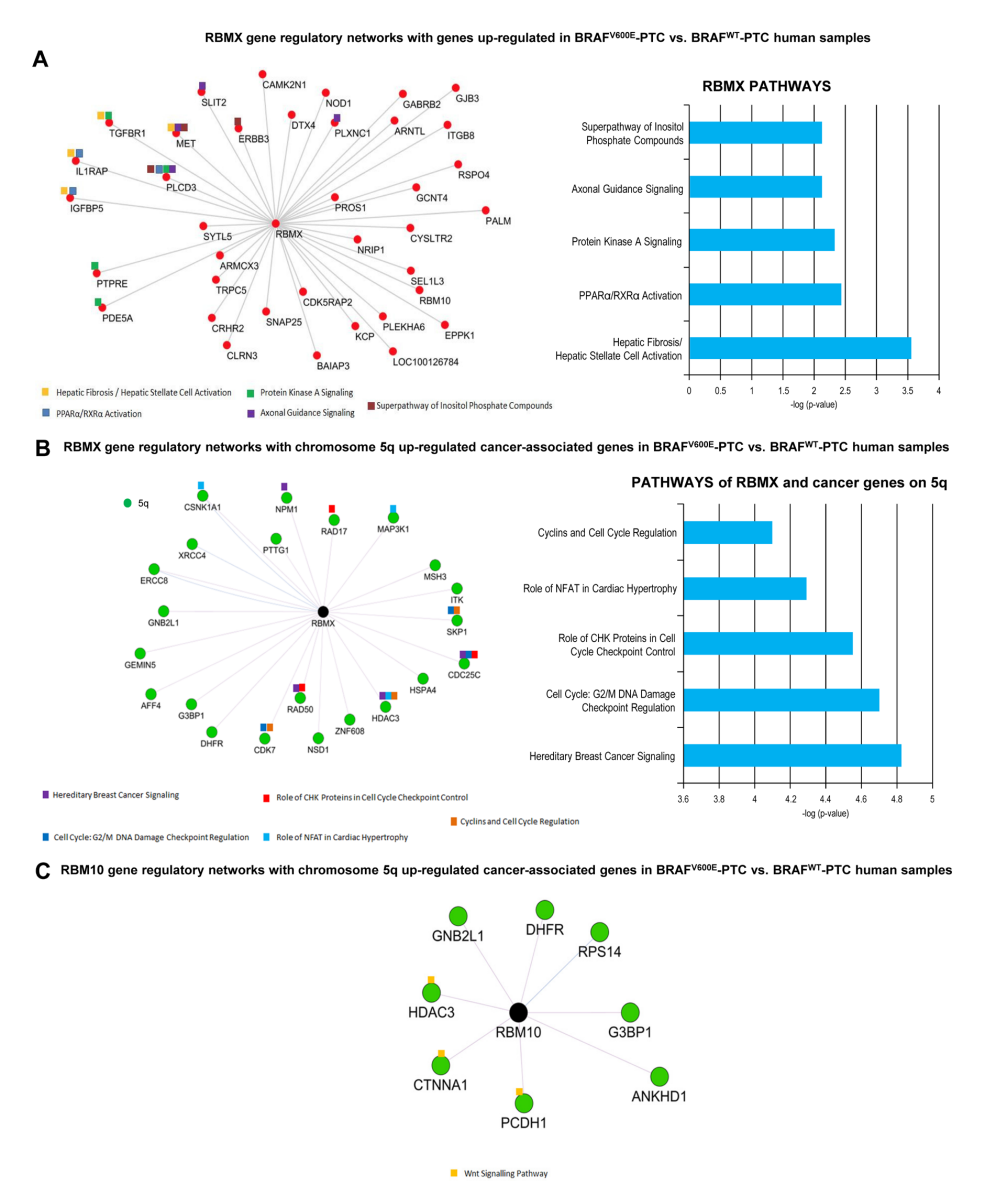
RBM genes regulatory networks with up-regulated genes in BRAF^V600E^-PTC versus BRAF^WT^-PTC human samples **A.** Differential gene expression analysis on BRAF^V600E^-PTC vs. BRAF^WT^-PTC human samples (PTC TCGA data base) identified 1884 genes with *p* < 0.05 and fold change 2. The co-expression analysis for these genes was performed on the basis of PTC TCGA data (interactions with *p* value <0.05 from correlation test were considered significant) to generate a map of RBMX and RBM10 gene regulatory networks. Image shows genes that depict significant interaction with RBMX top pathways of interactive genes regulatory networks. Red circles indicate up-regulated genes in BRAF^V600E^-PTC vs. BRAF^WT^-PTC samples. **B.** RBMX gene regulatory networks with chromosome 5q cancer-associated genes annotated by NCBI using BRAF^V600E^-PTC vs. BRAF^WT^-PTC human samples (PTC TCGA data base). The co-expression analysis for these genes was performed on the basis of PTC TCGA data (interactions with *p* value <0.05 from correlation test were considered statistically significant). For pathways analysis: -Log *p* value 1.3= *p* value= 0.05; -log *p* value 2= *p* value= 0.01; -log *p* value 3= *p* value= 0.001; -log P value 4= *p* value=0.0001. **C.** RBM10 gene regulatory networks with chromosome 5q cancer-associated genes annotated by NCBI using BRAF^V600E^-PTC vs. BRAF^WT^-PTC human samples (PTC TCGA data base) (interactions with *p* value <0.05 from correlation test were considered statistically significant).

In order to understand which pathways are connected with wild-type RBM genes in thyroid cancer, we first randomly built RBMX and RBM10 gene regulatory network (fold-change cutoff: 2; *p*-value <0.05) using human PTC samples from TCGA (The Cancer Genome Atlas). We found that only RBMX gene was significantly co-expressed with 38 out of 1884 (2%) up-regulated genes (Supplementary Table 5) in BRAF^V600E^-PTC compared to BRAF^WT^-PTC TCGA samples (Figure 5A). Those 38 genes are located on different chromosomes (Supplementary Table 5). Only 10 out of 38 (26.3%) genes interacted with RBMX creating regulatory networks which converge on crucial pathways for tumor microenvironment/ECM remodeling functions, cell polarity, inflammation and immune suppression processes, tumor survival, tumor metabolism, cell cycle regulation, and DNA damage-response (Figure 5A). Two out of 38 (5.2%) up-regulated genes are located on chr.5q (Supplementary Table 5) and play more specialized biological roles: brain functions and endosome-to-lysosome trafficking of membrane (i.e. GABRB2), and regulation of protein metabolism for glycosylation (i.e. GCNT4). Moreover, since our findings showed amplification of chromosome 5 we built RBMX and RBM10 gene regulatory network specifically with the cancer-associated genes located on chromosome 5p (33 genes) (Supplementary Table 6) and 5q (68 genes) (Supplementary Table 7) in BRAF^V600E^-PTC compared to BRAF^WT^-PTC TCGA samples. Twenty-two out of 1884 (1.1%) were chr.5q differentially expressed cancer-associated genes significantly up-regulated in BRAF^V600E^-PTC compared to BRAF^WT^-PTC and co-expressed with RBMX. Importantly, 9/22 (41%) of cancer-associated up-regulated genes on chromosome 5q significantly (*p* < 0.05) interacted with RBMX gene (Figure 5B), whereas none of the cancer genes on 5p (Supplementary Table 6) interacted with RBMX, indicating network specificity. Moreover, the pathways emerging from networks between RBMX and 5q cancer genes were involved in tumor survival and metabolism, G2 and mitosis homeostasis, cell cycles checkpoints regulation, chromosomes stability, DNA damage-response checkpoints, microenvironment homeostasis, extracellular matrix (ECM) organization, and regulation of MAPK signaling (Figure 5B). RBM10 had networks with 8 out of 1884 (0.4%) differentially expressed cancer-associated genes on 5q. These genes also resulted significantly up-regulated in BRAF^V600E^-PTC compared to BRAF^WT^-PTC and co-expressed with RBM10. Three out of 8 (37.5%) genes created significant regulatory networks with RBM10 evoking interconnected WNT signaling-related pathway (Figure 5C).

### Combined therapy with vemurafenib plus palbociclib overcomes primary and secondary resistance in BRAF^V600E^-positive and P16^-/-^ PTC patient-derived cells

**Figure 6:**
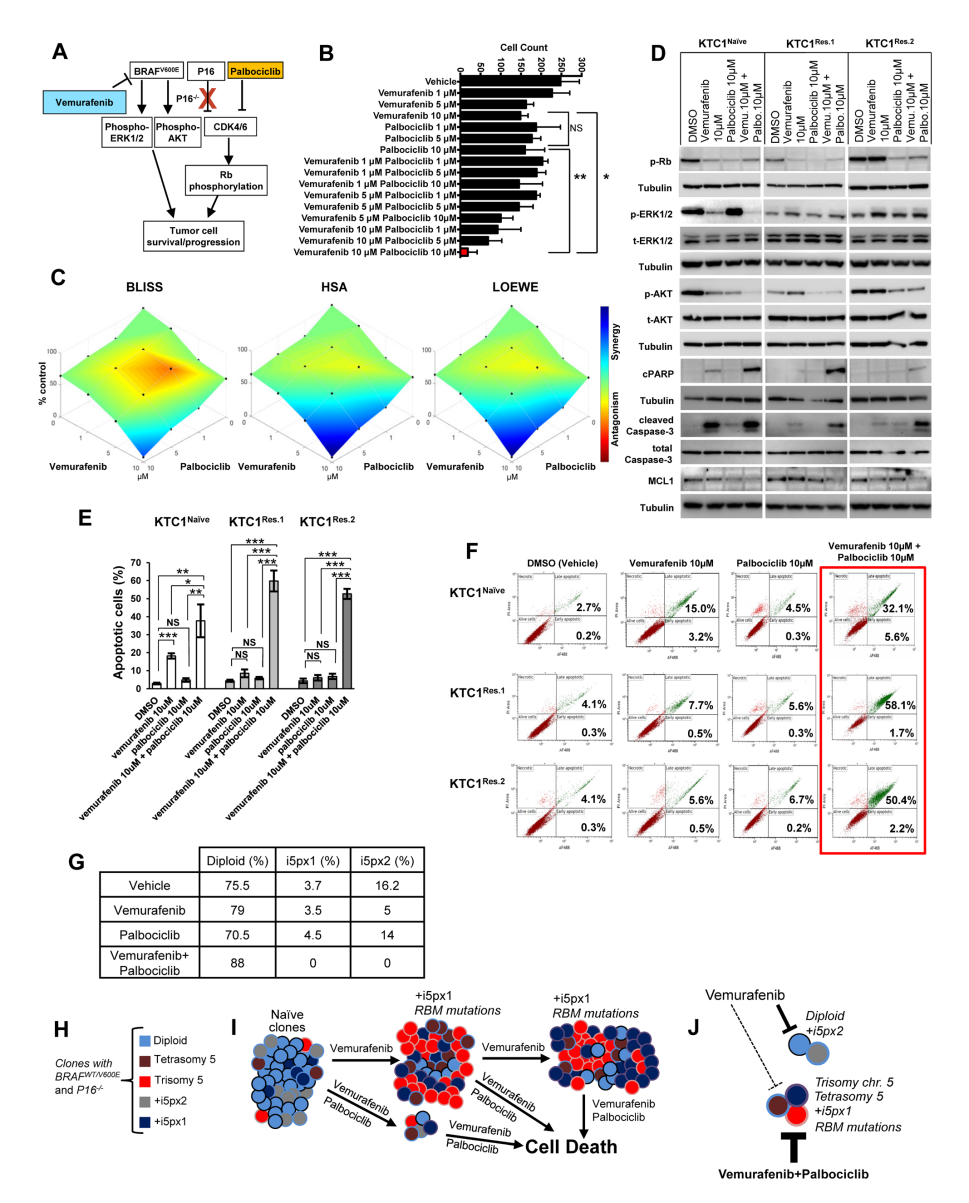
Combined therapy with vemurafenib plus palbociclib overcomes resistance to single agent treatments in PTC patient-derived cells with BRAF^V600E^ and P16^-/-^ **A.** Diagram of a proposed targeted therapy strategy in human invasive thyroid carcinoma cells harboring the heterozygous BRAF^V600E^ mutation and with P16 loss (P16^-/-^). **B.** Histogram shows cells number upon treatments with vehicle, vemurafenib, palbocilib, or combined therapy vemurafenib plus palbociclib; quantitative analysis was performed at 48 hours post-treatments by direct counting of adherent cells. These data represent the average ± standard deviation (error bars) of three replicates from two independent measurements. Statistical significance (*p <0.05; **p < 0.01; NS=not significant) was determined by one-way analysis of variance (ANOVA) using Tukey’s correction for multiple comparisons. **C.** Visualization of drug combinations: surface plots of naïve KTC1 cells treated vehicle, vemurafenib, palbocilib, or combined therapy vemurafenib plus palbociclib. Each point represents the mean of three replicates from two independent measurements. Plots were generated using Combenefit script by MATLAB R2017a by applying three methods for combined treatments: Bliss (effect-based approach); Highest Single Agent (HSA) (effect-based approach); and Loewe (dose-effect based approach). Level of antagonism or synergism is rapresented by color scale bar. **D.** Western blotting analysis of proteins expression levels at 24 hrs treatment with DMSO (vehicle), vemurafenib, palbocilib and combined therapy with vemurafenib plus palbocilib. These results were validated by two independent experiments. **E.** Quantification of apoptosis by annexin V and propidium iodide (PI) dual staining assay in KTC1^Naive^, KTC1^Res.1^ and KTC1^Res.2^ cells at 48 hrs treatment with DMSO (vehicle), 10 µM vemurafenib, 10 µM palbocilib and combined therapy with 10 µM vemurafenib + 10 µM palbocilib. These data represent the average ± standard deviation (error bars) of two independent replicate measurements (*p < 0.05, **p < 0.01, ***p < 0.001, NS=not significant). **F.** Representive Annexin V/PI Flow cytometry analysis of KTC1^Naive^, KTC1^Res.1^ and KTC1^Res.2^ cells at 48 hrs treatment with DMSO (vehicle), 10 µM vemurafenib, 10 µM palbocilib and combined therapy with 10 µM vemurafenib + 10 µM palbocilib. These data are representative of three independent replicate measurements (*p < 0.05, **p < 0.01, ***p < 0.001). **G.** Quantification of cell clones with either diploid or chromosome 5 aberrations assessed by interphase fluorescence *in situ* hybridization (FISH) analysis in KTC1^Naive^ cells treated for 48 hrs with vehicle, 10 µM vemurafenib (vemu), 10 µM palbociclib (palbo) or 10 µM vemurafenib plus 10 µM palbociclib. These results were validated quantifying 200 cells by two independent replicate measurements. **H.** KTC1 cells harboring the BRAF^V600E^ mutation and with P16 loss show heterogeneous clones which are diploid, aneuploidy (trisomy of chromosome 5, with one copy (+i5px1) or two copies (+i5px2) of isochromosome 5p), or with tetrasomy of chromosome 5 due to tetraploidy. **I.** Clonal selection and expansion of KTC1 cells during sustained treatment with vemurafenib; these selected clones acquire mutations in the RBM genes (e.g. RBMX, RBM10). **J.** Scheme of treatments with vemurafenib and palbociclib: dashed lines indicate that KTC1 cells clones with trisomy or tetrasomy of chromosome 5, +i5px1, or RBM mutations are resistant and less responsive to the single agent treatments. Whereas combined therapy with vemurafenib plus palbociclib (thick bold lines) significantly induces cell death and overcomes resistance to the single agent treatments by reducing clonal expansion of KTC1 cells with chromosome 5 aberrations.

BRAF^V600E^-positive PTC patient-derived KTC1 cells showed deletion of the P16 gene (P16^-/-^) (Figure 1B-1D). To determine if the inhibition of BRAF^V600E^ and targeting of CDK4/6 (P16 downstream effector) was effective against these tumor cells, we tested a therapeutic approach using vemurafenib plus palbociclib treatment (Figure 6A), two FDA approved drugs for metastatic BRAF^V600E^-positive melanoma and metastatic breast cancer, respectively. In order to assess the most effective doses of combined therapy with vemurafenib plus palbociclib, we treated thyroid tumor cells for 48 hours with a matrix of nine different drug doses combinations (Figure 6B, Supplementary Figure 3). Our results showed that 10 µM vemurafenib plus 10 µM palbociclib significantly provided the best therapeutic efficacy against thyroid tumor cells (Figure 6B). This combined therapy significantly decreased cells number of 13.3, 8 and 8.6 fold-change compared to vehicle, vemurafenib or palbociclib treatments, respectively (Figure 6B, Supplementary Figure 3), likely by synergistic effects (Figure 6C). Importantly, we found significant induction of apoptosis in both vemurafenib-naïve and vemurafenib-resistant cells when we used this combined therapy as shown by annexin V/PI apoptosis analysis (Figure 6E-6F) and by the substantial reduction of pro-survival factors such as phospho(p)-Rb, pAKT and MCL1 and increase of cell death markers (cleaved caspase-3 and cleaved PARP, Figure 6D). More importantly, this combined therapy was significantly effective to cause apoptosis compared to either single agents or vehicle-treated cells (Figure 6E). Additionally, we tested the therapeutic efficacy of these drugs in advanced/aggressive ATC-derived cells. ATC is a deadly disease with a dismal prognosis. ATC cells harbored BRAF^V600E^ and showed wild-type P16 status (Supplementary Figure 4). They were also effectively sensitive to vemurafenib plus palbociclib treatment which caused a significant reduction of cell number over the time (Supplementary Figure 5), suggesting that this therapy can be also used in BRAF^V600E^-positive ATC with wild-type P16. We also found that vemurafenib plus palbociclib maintained down-regulation of p-AKT (critical pro-survival factor) protein levels when RBMX (sh-RBMX) was silenced compared to the single agent or vehicle treatments (Figure 4B), suggesting that this combined therapeutic approach could have anti-tumor survival activity by affecting AKT expression levels and subsequently its downstream effectors. Critically, this combined therapy substantially reduced the number of BRAF^V600E^-positive and P16^-/-^ tumor cell clones with chromosome 5 aberrations (i.e. extra copies of +i5p) compared to vehicle-treated cells (from 3.7% or 16.2% to 0) or more importantly to single agent treatments within 48 hrs (Table in Figure 6G). Whereas single agent treatments did not show robust changes in +5px1 copies, vemurafenib treatment decreased +i5px2 extra copies compared to vehicle-treated cells (Table in Figure 6G). Collectively, our findings indicate that vemurafenib treatment on BRAF^V600E^-positive invasive thyroid carcinoma cells with P16^-/-^ acts as a selective pressure favoring the appearance of resistant clones with chromosome 5 aberrations and RBM genes (e.g. RBMX and RBM10) *de novo* mutations. Importantly, combined inhibition of BRAF^V600E^ and CDK4/6 synergistically induces apoptosis in both naïve and resistant tumor cells, indicating that the mechanism of resistance to vemurafenib requires CDK4/6 pathway activation. In summary, this combined therapeutic approach results in both effective early treatment preventing the selection and expansion of aberrant thyroid tumor cell clones with primary resistance, but also as late intervention on vemurafenib resistant tumors with secondary resistance (Figure 6H-6J).

## DISCUSSION

PTC is associated with a favorable long term survival. However, the subgroups of patients who develop distant metastases have a worse prognosis [30]. BRAF^V600E^ can be measured in the blood of patients with metastatic PTC (e.g. lung metastasis) [31]. Patients with advanced BRAF^V600E^-PTC generally showed partial response to BRAF^V600E^ inhibitors [32]. Phase I study of vemurafenib in three patients with BRAF^V600E^-PTC reported that one had partial response with reduction of pulmonary target lesions by 31%, and the duration of response was 7.6 months before the disease progressed in the lungs and bones [33]. Therefore, advanced PTC showed partial response to vemurafenib and unavoidably relapses as refractory carcinomas. Unfortunately, the majority of patients with cancer may develop resistance in a short time from the initiation of therapy. How metastatic PTC becomes resistant to BRAF^V600E^ inhibitors (e.g. vemurafenib) remains poorly understood. In this study, we have investigated the genetic properties of vemurafenib-resistant cells in a unique model of heterozygous BRAF^V600E^ human thyroid cancer cells established from a metastatic pleural effusion of a BRAF^V600E^-positive recurrent PTC patient. Tumor cells have genomic instability and often have complex karyotypes with numerous chromosomal rearrangements and polyploidy (e.g. aneuploidy, tetraploidy). However, KTC1 cells showed diploid and aneuploid/tetraploid clones with relatively few chromosomal aberrations, representing an ideal model to study clonal evolution and occurrence of drug resistance. KTC1 cells were exposed to vemurafenib to eradicate sensitive cell subpopulations and expand resistant clones. We found resistance to vemurafenib in tumor cells harboring amplification of chromosome 5 and mutations in RBM genes which are fundamental for genome stability during cell division [28]. These findings could indicate that treatment failure and disease relapse in metastatic advanced BRAF^V600E^-PTC might be linked to the selection of aneuploidy/tetraploid cell subpopulations with extra copies of chromosome 5. Interestingly, we found two independent *de novo* mutations in the RBM gene family (i.e. RBM10 and RBMX) in the vemurafenib-resistant cells but not in the vemurafenib-naïve thyroid tumor cells which did not show measurable allelic fractions for these mutations. These genes are involved in the regulation of RNA splicing [34] [35] and apoptosis [36]. RBM10 is a tumor suppressor that represses Notch signaling and cell proliferation [37]. Importantly, RBM10 has been recently found to be mutated in the aggressive and fatal subtypes of thyroid carcinoma, and the overall survival of patients with RBM10 mutations was significantly poorer compared to those with wild type RBM10 [38], suggesting a role for this gene in thyroid tumor aggressiveness and progression. RBMX is a regulator for maintenance and centromeric protection of sister chromatid cohesion during cell division and for chromosomes stability [28, 29]. The karyotype of cancer is highly variable and often shows genetic complexity with chromosomal instability (e.g. chromosomal loss or gain), ranging from hypodiploidy to tetraploidy, or to polyploidy. Tetraploidy or polyploidy are characterized by whole extra sets of chromosomes, and arise in different conditions including cancer [39-41]. Tetraploid cells can originate from mitotic arrest/slippage, cytokinesis failure, or cell-cell fusions which can result in genomic instability [41]. Importantly, we found an increased percentage of cells with chromosome 5 tetrasomy due to tetraploidy when RBMX was silenced during acute treatment with vemurafenib, suggesting that loss of function of this gene might play a crucial role in tetraploidization and exacerbates this *phenotype*. Also, the reduction of RBMX protein levels impacted on the down-regulation of phospho-AKT protein levels; these results might suggest the importance of RBMX protein not only to prevent DNA errors but also to inhibit AKT pathways which are fundamental to promote tumor cell survival and growth. However, further studies will be needed to determine mechanisms by which RBMX or RBM10 mutants affect chromosome 5 expansion and tetraploidization during treatment with BRAF^V600E^ inhibitors. Overall, our results suggest the development of complex processes during sustained treatment with BRAF^V600E^ inhibitors (i.e. vemurafenib) in the invasive spontaneously-immortalized PTC cells that we have exhaustively characterized: (i) initial presence of a heterogeneous tumor cell population including mainly diploid clones, but also aneuploid or tetraploid clones; (ii) gain of secondary mutations (i.e. in the RBM genes) permissive to chromosomal aberrations and tetraploidization; and (iii) selection and expansion of clones with advantageous aberrations (i.e. chromosome 5 trisomy, gain of 1 copy of isochromosome 5p or tetraploidy). The spectrum of isochromosomes differed among neoplasms, e.g. +i5p has been proposed as one potential mechanism to drive tumor initiation and progression [42]. Although molecular mechanisms underlying the formation of isochromosomes are not yet well understood, our findings linking clone expansion of cells harboring 1 copy of isochromosome 5p (i.e. +5px1) in vemurafenib-resistant tumor cells might suggest that gene amplifications can occur in the presence of long-term treatment with vemurafenib as pro-survival response to tumor cells. RBMX elicits gene regulatory networks with cancer-associated genes of chromosome 5q but not of 5p. These networks represent top pathways of synergy in genetic interactions that control cell cycle checkpoints in response to DNA-damage and mechanisms of chromosome stability. Intriguingly, we did not find changes in growth rate in the resistant cells compared to the naïve cells. Importantly, we found a significant increase of resistant cells in G2-M phase. Since we did not find an increased growth rate of vemurafenib-resistant cells, we hypothesized that some tumor cell populations slowed down or blocked in G2/M phase, with an accumulation in this phase of the cell cycle. Also, our cytogenetic analysis revealed that tetraploid cells increased when BRAF^V600E^ was inhibited by vemurafenib and RBMX was silenced. Collectively, these results suggest that vemurafenib-resistant cells enter cell cycle doubling their DNA content; however, they might not undergo to final cell division, resulting in an increase of tretraploidy. Loss of function of RBM genes (e.g. RBMX) and amplification of chromosome 5 genes in the vemurafenib-resistant cells might contribute to cell cycle checkpoint dysfunctions. Ultimately, these complex biological processes which are evoked in vemurafenib-resistant cells might enhance mitotic errors, leading to tumor progression. Generally, PTC shows low frequency in somatic copy-number alterations compared to somatic mutations [1]. We analyzed the PTC TCGA data set [1]; 5p showed a trend in increase of copy-number (0.2 cutoff) from stage I (0%) to stage III (3.5%) in BRAF^V600E^-PTC, and from 1.03% in stage I to 13.04% in stage III in BRAF^WT^-PTC. BRAF^WT^-PTC showed copy-number gain in 5q similarly as observed for 5p; whereas 5q copy number did not change in BRAF^V600E^-PTC across all tumor stages. This result suggests that amplifications of 5q might be a sub-clonal event in BRAF^V600E^-PTC during sustained therapy with BRAF^V600E^ inhibitors (e.g. vemurafenib). Vemurafenib has been recently used in patients with metastatic/refractory BRAF^V600E^-PTC and continuously administered twice a day in cycles of 28 days. This therapy showed anti-tumor activity with partial response in 10/26 (38.5%, best overall response) patients. Four patients (15%) died after a median follow-up of 18.8 months [43]. These clinical findings indicate that inhibition of BRAF^V600E^ is not effective in all metastatic BRAF^V600E^-PTC patients and might be provocative for PTC cells to evoke complex mechanisms which escape this targeted/precise therapy. Finding mutations which change the wild-type functions in two genes which belong to the same family (i.e. RBMX and RBM10) and are involved in chromosomal segregation suggests that acquired secondary mutations may converge to allow or promote chromosome 5 copy number gain in resistant cells. This may be one of the possible mechanisms of resistance to BRAF^V600E^ inhibitors in metastatic BRAF^V600E^-PTC. KTC1 cells show also deletion of P16 loss which is frequent in many cancers and results in the over-activation of CDK4/6 axis, critical to cell cycle entry [44]. Aberrant regulation of the cell cycle is one of the crucial mechanisms in tumor progression [45], which includes deregulation of checkpoints from G1 phase until mitosis [44]. Preclinical studies have shown that CDK4/6 inhibitors induce G1 arrest of human cancer cell lines, suppressing tumor growth or inducing tumor regression [46]. We have applied a combined therapeutic approach using, for the first time to the best of our knowledge, BRAF^V600E^ and CDK4/6 inhibitors in human thyroid carcinoma cells. Remarkably, this combined therapy suppressed the clonal expansion of cells with aberrations of chromosome 5 (e.g. +i5px1). This treatment was able to induce apoptosis in both naïve and vemurafenib-resistant cells. Recently, it has been shown that vemurafenib-resistant melanoma cells were sensitive to the sustained exposure with palbociclib which determined cell senescence [47]. Also, combined therapy with BRAF^V600E^ and CDK4/6 inhibitors was effective to prolong survival in mouse models for astrocytoma [48]. Although further studies will be necessary, our results suggest that targeting BRAF^V600E^ and CDK4/6 in aggressive thyroid cancer can trigger apoptosis by overcoming CDK4/6-dependent cell cycle checkpoints dysfunctions which could allow or even lead to genomic instability. Genomic heterogeneity/instability might contribute significantly to BRAF^V600E^ inhibitor treatment failure, suggesting that targeting different cancer-associated pathways can be strength for long duration therapeutic responses. We also believe that orthogonal therapeutic approaches during treatment are required and fundamental to kill the majority of tumor cells at first with combined therapies. Our therapeutic approach was able to overcome both primary and secondary resistance to single agent treatments in invasive human thyroid carcinoma cells. It has been recently shown that KRAS G12D mutation can also be a genetic mechanism of secondary resistance to vemurafenib in KTC1 cells. Authors using a different therapeutic strategy found that subpopulation of KTC1 cells acquired resistance to this drug. However, these resistant cells became less sensitive to combined therapy with vemurafenib plus MEK1 or AKT inhibitors [49]. Therefore, single agent therapeutic interventions might reduce tumor cells number, but inadvertently provides powerful selective pressure for the expansion of resistant sub-clones. Clonal heterogeneity within tumors can be considered a substrate for evolutionary adaptation to the environment via Darwinian selection. The capacity of some subpopulations of tumor cells to survive in different environments is at the base of metastasis establishment and drugs resistance. Genetic divergence occurs during tumor progression, however, when selective pressures (e.g. drug treatments) are used, convergent evolution might occur. Our study is limited by the lack of human samples from clinical trials of patients with metastatic BRAF^V600E^-PTC treated with BRAF^V600E^ inhibitors; however, our results are substantiated by our integrated *ex-vivo* approaches and applications of PTC TCGA patient-derived. It is also possible that transduction of RBMX or RBM10 mutants in vemurafenib-naïve PTC patient-derived cells might elicit more potent phenotypes than shRNA-mediated loss-of-function of the RBMX^WT^.

In summary, we report for the first time that during vemurafenib therapy human invasive thyroid tumor cells with BRAF^V600E^ and P16 loss acquired secondary resistance to this BRAF^V600E^ inhibitor by amplification of chromosome 5, gain of the isochromosome 5p, tetrasomy 5 due tetraploidy, and *de novo* mutations in the RBM genes family. Critically, our findings are translational and suggest that combined treatment with BRAF^WT/V600E^ and CDK4/6 inhibitors could represent a novel therapeutic strategy to treat vemurafenib-resistant or vemurafenib-naïve BRAF^WT/V600E^-PTC refractory to standard therapies. This combined therapy with vemurafenib plus palbociclib results in a very efficient strategy to target tumor cells either with primary or secondary resistance. The stable loss of P16 function in the background could represent a constraint that positively selects cell clones resistant to vemurafenib treatment due to the activation of P16 downstream effectors such as CDK4/6. Our findings support the proof of concept that this combined therapy can be tested in a clinical trial of patients with metastatic BRAF^WT/V600E^-PTC or also with BRAF^WT/V600E^-ATC.

## MATERIALS AND METHODS

### Cell cultures

We used KTC1 (BRAF^WT/V600E^), TPC1 (BRAF^WT/WT^), 8505c (BRAF^V600E^) and SW1736 (BRAF^WT/V600E^) human thyroid carcinoma cell lines. KTC1 is a spontaneously immortalized human thyroid carcinoma cell line which harbors BRAF^WT/V600E^ mutation. It was established from the metastatic pleural effusion from a recurrent and radioiodine (RAI) refractory PTC in a 60-year-old male patient [50] by Dr. J. Kurebayashi (Department of Breast and Thyroid Surgery Kawasaki Medical School Kurashiki, Japan) and provided by Dr. Rebecca E. Schweppe (University of Colorado, USA). More details are reported in the Supplementary Methods.

### Drugs treatments

Vemurafenib (PLX4032, RG7204) (Selleckchem, Houston, TX, USA) was dissolved according to manufacturer’s instructions in 100% dimethyl sulfoxide (DMSO, vehicle) (Sigma, USA) to achieve a stock concentration of 10 mM for *in vitro* assays. Palbociclib also named PD0332991 HCl (Selleckchem, USA) is an inhibitor of CDK4/6. Powder was dissolved in 100% DMSO (Sigma, USA) according to the manufactures instructions preparing 5 mM stocks. Intermediate doses of vemurafenib or palbociclib were prepared in 100% DMSO and diluted in 0.2% FBS high glucose DMEM in order to achieve the desired final concentrations maintaining a constant final concentration at 2% DMSO for optimal solubility (more details are reported in the Supplementary Methods). Combined treatments of vemurafenib plus palbociclib were calculated using Combenefit script [51] by MATLAB R2017a applying Bliss, Highest Single Agent (HSA), and Loewe methods in order to assess drug synergy and antagonism. Cells were treated for 48 hours in the presence of 0.2% FBS high glucose DMEM at final 2% DMSO with: 1, 5 or 10 µM of either vemurafenib or palbociclib; or combined therapy with vemurafenib plus palbociclib combining all above doses. Vehicle was used as untreated control (2% DMSO diluted in 0.2% FBS high glucose DMEM). Before adding treatments, cells were washed with PBS from 10% FBS high glucose DMEM. Quantitative analysis was performed by direct count of adherent cells (magnification: 10×). Vehicle (control) was 2% DMSO diluted in 0.2% FBS high glucose DMEM. Data were plotted applying a matrix of cell count using GraphPad Prism 6.

### Model of resistance to vemurafenib therapy

In order to select KTC1 cells resistant to BRAF^V600E^ inhibitor (vemurafenib), cells were treated with 10 µM vemurafenib for 12 (10 cycles, KTC1^Res.1^ cells) or 24 (20 cycles, KTC1^Res.2^ cells) weeks. Cycle of therapy is defined as follows: treatment of cells with 10 µM vemurafenib in the presence of 0.2% FBS high glucose DMEM until 90% were dead cells and then recover them in 10% FBS high glucose DMEM without vemurafenib.

### Exome sequencing

Mutation analysis for single nucleotide variants (SNV) was performed using MuTect v1.1.4 and annotated by Variant Effect Predictor (VEP) [52, 53]. Variants represented at >1% in either the African-American or European-American and not in COSMIC > 2x were considered to be likely germline and filtered out on the SNV/indel report. Somatic genetic variants which showed an allelic fraction greater than 30% were considered for variant calling passing criteria to exclude technical artifacts, including removal of variants located at the last mapped base (or outside) of amplicon target regions and variants with the majority of supporting reads harboring excess additional mismatches or indels (likely sequencing error). More details are reported in the Supplementary Methods.

### Cytogenetic analysis

Cytogenetic analysis of metaphases was performed on cultured thyroid carcinoma cell lines (KTC1^Naive^, KTC1^Res.1^, KTC1^Res.2^, and SW1736) using standard techniques (Beth Israel Deaconess Medical Center, Boston, MA). GTG (G-banding by Trypsin treatment followed by Giemsa stain) banded metaphases were obtained using established harvesting and banding techniques.

### Fluorescence *in situ* hybridization

Fluorescence *in situ* hybridization (FISH) was performed with Abbott Molecular probes (Abbott Molecular, Des Plaines, IL) following the manufacturer’s recommended protocol. 200 interphase cells were scored for each probe hybridization for each thyroid carcinoma cell line.

### Affymetrix CytoScan HD oligonucleotide-SNP microarray

Affymetrix CytoScan HD array (Affymetrix, Santa Clara, CA) was used to identify chromosomal gains and losses and their breakpoints using DNA from the same thyroid carcinoma cell cultures used in the cytogenetic analysis. Microarray analysis was performed according to the manufacturer’s protocol. Each sample was digested with *Nsp*I, ligated to adaptors, and amplified with PCR. The PCR product was purified using a magnetic separation technique, fragmented, and labeled before hybridization to the microarray. The oligo-SNP (single nucleotide polymorphism) array contains approximately 2.67 million probes, including 1.9 million copy number probes and 750,000 SNP probes. Copy number alterations were analyzed using Chromosome Analysis Suite (ChAS) software (Affymetrix). The gene content of the duplicated and deleted regions was obtained from the UCSC Genome Browser and the NCBI database.

### Gene regulatory networks and pathways analysis in human PTC samples

We downloaded clinical and RNA-seq data of PTC from TCGA website and used edgeR package in R to get differentially expressed genes using 234 BRAF^V600E^-PTC and 255 BRAF^WT^-PTC samples. Genes with p-value less than 0.05 were considered differentially expressed and statistically significant. WGCNA (weighted correlation network analysis) was used to build a network between differentially expressed genes obtained from BRAF^V600E^-PTC versus BRAF^WT^-PTC TCGA on the basis of coexpression information of genes. More details are reported in the Supplementary Methods.

### Statistical analysis

Statistical analysis was carried out using GraphPad Prism 6 software (San Diego, CA, USA), SAS/STAT(R) 9.2, and Microsoft Excel (Boston, MA, USA) statistical tools. T-student, one-way analysis of variance (ANOVA) using Tukey’s correction for multiple comparisons, Fisher’s exact, slope analysis (angular coefficient, m-value), Mantel-Haenszel (M-H), and chi-square tests were used. Data are reported as the averaged value, and error bars represent the standard deviation of the average for each group. Results with *p* values below 0.05 were considered statistically significant.

We also used western blotting, cell cycle, cell viability, cell growth, apoptosis, and viral transduction (for gene knockdown) assays; and gene regulatory networks/pathways analyses (for more details, see the Supplementary Methods).

## SUPPLEMENTARY MATERIALS FIGURES AND TABLES
















