# Supplementary material for: Vemurafenib-resistance via de novo RBM genes mutations and chromosome 5 aberrations is overcome by combined therapy with palbociclib in thyroid carcinoma with BRAFV600E
Source: Oncotarget. 2017 Sep 24;8(49):84743–60. doi: 10.18632/oncotarget.21262 (PMC5689570; doi:10.18632/oncotarget.21262)
Supplement: Supplementary file 1 [file oncotarget-08-84743-s001.pdf]

Vemurafenib-resistance via de novo RBM genes mutations and chromosome 5 aberrations is overcome by combined therapy with palbociclib in thyroid carcinoma with $BRAF^{V600E}$

SUPPLEMENTARY MATERIALS

Cell cultures

We used KTC1, TPC1, 8505c and SW1736 human thyroid carcinoma cell lines. KTC1 is a spontaneously immortalized human thyroid carcinoma cell line which harbors the heterozygous $BRAF^{WT/V600E}$ mutation. It was established from the metastatic pleural effusion from a recurrent and radioiodine (RAI) refractory papillary thyroid carcinoma (PTC) in a 60-year-old male patient [1] by Dr. J. Kurebayashi (Department of Breast and Thyroid Surgery Kawasaki Medical School Kurashiki, Japan) and provided by Dr. Rebecca E. Schweppe (University of Colorado, USA). TPC-1 cell line (PTC-derived, harboring RET/PTC-1 and with $BRAF^{WT}$) was provided by Dr. F. Frasca (University of Catania, Italy). The 8505c (anaplastic/ undifferentiated thyroid carcinoma (ATC)-derived) cell line harbors the hemizygous/homozygous $BRAF^{V600E}$ mutation was purchased from DSMZ (German collection of microorganisms and cell culture, Braunschweig, Germany) [2] [3] [4]. SW1736 is an ATC cell line harboring the heterozygous $BRAF^{WT/V600E}$ mutation. Human thyroid carcinoma cells were validated by genomic/genotyping analyses (e.g. $BRAF^{V600E}$ mutation analysis) and were grown in high glucose DMEM (Corning, USA) medium supplemented with 10% FBS (fetal bovine serum, Atlanta Biologicals cat#S11550, Atlanta, GA, USA) and plus ampicillin/streptomycin antibiotic and antimycotic solution (Corning, USA). During *in vitro* assays cells were grown in high glucose DMEM growth medium supplemented with 0.2% FBS. The human embryonic kidney (HEK) 293T cell line was kindly provided by Dr. Laura E. Benjamin and grown in DMEM supplemented with 10% FBS and plus ampicillin/ streptomycin antibiotic and antimycotic solution 100× (Corning, USA).

Vemurafenib

Vemurafenib (PLX4032, RG7204) (Selleckchem, Houston, TX, USA) was dissolved according to manufacturer instructions in 100% dimethyl sulfoxide (DMSO, vehicle) (Sigma, USA) to achieve a stock concentration of 10 mM for *in vitro* assays. Ten mM was diluted to 2 mM in 100% DMSO; then, intermediate doses of vemurafenib were prepared in 100% DMSO; finally intermediate doses were diluted in 0.2% FBS high glucose DMEM in order to achieve the desired final concentrations of 0.5 µM, 1 µM, 5 µM, and 10 µM vemurafenib maintaining a constant final concentration at 2% DMSO for optimal solubility. Vehicle was used as untreated control (2% DMSO diluted in 0.2% FBS high glucose DMEM). Before adding treatments, cells were washed with PBS from 10% FBS high glucose DMEM.

Palbociclib

Palbociclib also named PD0332991 HCl (Selleckchem, USA) is an inhibitor of CDK4/6. Powder was dissolved in 100% DMSO (Sigma, USA) according to the manufactures instructions preparing 5 mM stocks. Intermediate doses of palbociclib were prepared in 100% DMSO; finally, intermediate doses were diluted in 0.2% FBS high glucose DMEM in order to achieve the desired final concentrations of 1 µM, 5 µM, and 10 µM palbociclib maintaining a constant final concentration at 2% DMSO for optimal solubility.

Western Blotting

Cells were grown in 10-cm dishes in 10% FBS high glucose DMEM. They were treated with vehicle (DMSO), vemurafenib, palbociclib or comnined therapy with vemurafenib plus palbociclib in the presence of 0.2% FBS high glucose DMEM when reached about 90-100% confluence. Cells were lysed at the indicated time points. Western blotting assays were performed according to a standard procedure, and the protein lysis buffer was composed of 10 mM Hepes (pH 7.40), 150 mM NaCl, 5 mM EDTA, 1 mM EGTA, 1 mM sodium vanadate, 5 mM sodium flouride, and 1% Triton X-100; protease and

phosphatase inhibitors (Thermo Fisher Scientific, MA, USA) were used for protein extractions [3]. The bands intensity was quantified by densitometry analysis (ImageJ software, USA). We used the following antibodies: tubulin (cat#T9026, Sigma-Aldrich, USA), actin-HRP (Santa Cruz, USA), phospho-ERK1/2 (cat#9101, Cell Signaling, USA), total-ERK1/2, (cat#9102, Cell Signaling, USA), RBMX (cat#14794, Cell Signaling, USA), phospho-AKT (cat#4060, Cell Signaling, USA), total-AKT (cat#9272, Cell Signaling, USA), cleaved PARP (cat#5625, Cell Signaling, USA), cleaved caspase 3 (cat#9664, Cell Signaling, USA), total caspase 3 (cat#9662, Cell Signaling, USA), phospho-Rb (cat#9301, Cell Signaling, USA), and MCL1 (cat#32087, Abcam, USA).

Growth curves

KTC1 cells were seeded in 6-well plates and grown in 10% FBS high glucose DMEM until reaching 50% cell density. Treatments were applied as indicated in the methods section ‘drugs treatments’. Cell growth curves were determined by direct counting of adherent cells: cells were imaged (magnification: 10×) at the indicated time points up to 7 days by EVOS microscope (Life Technologies, MA, USA) and quantified with cell counter function of ImageJ (ImageJ software, USA).

Assessment of vemurafenib and palbociclib doses for combined treatment

In order to assess the most effective doses of combined therapy with vemurafenib plus palbociclib, KTC1 cells were seeded in the 24-well plates (one plate per each time point) and treated when they did reach out 100% cell density per well. Cells were treated for 48 hours in the presence of 0.2% FBS high glucose DMEM with: 1, 5 or 10 μM of either vemurafenib or palbociclib; or combined therapy with vemurafenib plus palbociclib combining all above doses. Control cells were treated with vehicle (2% DMSO). The different conditions were imaged and quantified as for growth curves by counting (magnification: 10×) adherent cells at 48 hrs of treatment. The most effective combined therapy was quantified by sorting the cells number from higher to lower values based on the number of adherent cells. Data were plotted as matrix of cell count using GraphPad Prism 6.

Cell cycle analysis

Cells were seeded at about 90% confluence in 6-well dishes and grown in 10% FBS high glucose DMEM. After 24 hrs, cells were treated with 10 μM vemurafenib, 10 μM Palbociclib, combined therapy with 10 μM vemurafenib plus 10 μM palbociclib, or vehicle (2% DMSO) for 48 hours in the presence of in 0.2% FBS high glucose DMEM. After 45 hours cells were pulsed with 10 μM BrdU for 3 hours, then, adherent cells were trypsinized and pelleted with the supernatant. Cell pellets were fixed in pre-chilled (−20°C) ethanol 75%, pelleted at 400 × g at room temperature, suspended in 0.5% PBS/BSA, incubated in 2M HCl 0.5% BSA for 20 min at room temperature, washed with 0.5% PBS/BSA, and centrifuged 5 minutes at 400 × g at room temperature. The cells were suspended in 0.1M sodium borate (pH 8.5) for 2 min at room temperature, washed with 0.5% PBS/BSA, and centrifuged 5 min at 400 × g at room temperature. Cells were pelleted and washed twice with 0.5% PBS/BSA. For the BrdU staining, cell samples were suspended according to the manufacturer’s instructions in a PBS 0.5% Tween-20 solution containing anti-FITC–conjugated BrdU antibody (BD Biosciences). Cells were pelleted and washed twice with 0.5% PBS/BSA. Finally, cell pellets were suspended in 500 μL of Propidium iodide (Thermo Fisher, MA, USA) solution to a final concentration of 10 μg/mL plus RNase 10 mg/mL (Sigma) and incubated at room temperature for 30 minutes. Stained cells were analyzed by flow cytometry using a FACS Gallios (Beckman Coulter, Miami, FL, USA).

Apoptosis assay

Cells were seeded at about 90% confluence in 6-well dishes and grown in 10% FBS high glucose DMEM. After 24 hrs, cells were treated with 10 μM vemurafenib, 10 μM Palbociclib, combined therapy with 10 μM vemurafenib plus 10 μM palbociclib, or vehicle (2% DMSO) for 48 hours in the presence of 0.2% FBS high glucose DMEM. After 48 hours, adherent cells were trypsinized and pelleted with the supernatant. Cell pellets were fixed at 4 °C with 75% ethanol. Cell death was assessed using annexin V Alexa Fluor 488 and Propidium iodide double staining (Life Technologies, MA, USA) by flow cytometry analysis (FACS Gallios, Beckman Coulter, Miami, FL, USA) according to the manufactures instructions.

Silencing technique

RBMX and helper plasmids [5] were kindly provided from Dr. Stephen J. Elledge (Department of Genetics, Harvard Medical School, Boston). Vector code #BA111 was used as control (sh-FF); vector code #BA103 (sh-RBMX, description: V3LHS_339669) and #BA106 (sh-RBMX, description: V3LHS_645229) in pMSCV-PM were used for RBMX knockdown. Viral transductions were performed as follows: HEK 293T cells were seeded in 10-cm dishes and cotransfected the next day with each virus and helper plasmids. Media with progeny virus from transfected HEK 293T cells were collected 48 hours later and filtered with 0.45 µm filters (Millipore, USA) and freshly used to transduce KTC1 cells for either 5 or 24 hours in the presence of 8 µg/mL polybrene (Sigma). Finally, stable transduced KTC1 cells with viral vectors were selected with puromycin (Sigma-Aldrich). Efficiency of RBMX knockdown in the KTC1 cells was assessed by western blotting. All assays were performed in duplicate. We used the most efficient shRNA (vector code #BA103, V3LHS_339669) among the two tested.

Exome sequencing

From each sample, 100 ng of genomic DNA was quantified using the Quant-iT Picogreen dsDNA assay kit (Invitrogen, Carlsbad, CA), randomly fragmented to an average size of 250bp using a Covaris S2 instrument (Covaris Inc, Woburn, MA) and purified using Agencourt AMPure XP beads (Beckman Coulter, Danvers, MA) according to manufacturer's guidelines. Size-selected DNA was then ligated to specific adaptors during library preparation using the Kapa Hyper prep kit (#KK8504, Kapa Biosystems, Inc, Woburn, MA), and the size range was confirmed using an Agilent Bioanalyzer (Agilent Technologies, Santa Clara, CA). Libraries were quantified and normalized based on the number of reads after a MiSeq sequencing run (Illumina Inc, San Diego, CA) and pooled with one other library to a total of 750 ng for exon enrichment using the SureSelect Human All Exon V5 baits (Elid: S04380110, Agilent Technologies, Santa Clara, CA) enriched with a custom set of introns for translocation detection. All captures were then pooled and sequenced at 100bp paired-end on 1 lane of a HiSeq3000 (Illumina Inc, San Diego, CA). After sequencing, pooled sample reads were de-convoluted (de-multiplexed) and sorted using the Picard tools [6] [7]. Reads were aligned to the reference sequence b37 edition from the Human Genome Reference Consortium using BWA using the following parameters "-q 5 -l 32 -k 2 -o 1" and duplicate reads were identified and removed using the Picard tools. The alignments were further refined for localized realignment around indel sites and recalibration of the quality scores was also performed using GATK tools [7, 8]. Mutation analysis for single nucleotide variants (SNV) was performed using MuTect v1.1.4 and annotated by Variant Effect Predictor (VEP) [9, 10]. We used the Somatic Indel Detector tool that is part of the GATK for indel calling. Consecutive variants in the same codon were re-annotated to maximize the effect on the codon and marked as "Phased" variants. MuTect was run in paired mode using CEPH1408 as the "project normal." In addition, each variant was annotated with its population frequency using the 6,500 exome release of the Exome Sequencing Project (ESP) database. Variants represented at >1% in either the African-American or European-American and not in COSMIC > 2x were considered to be likely germline and filtered out on the SNV/indel report. Somatic genetic variants which showed an allelic fraction greater than 30% were considered for variant calling passing criteria to exclude technical artifacts, including removal of variants located at the last mapped base (or outside) of amplicon target regions and variants with the majority of supporting reads harboring excess additional mismatches or indels (likely sequencing error).

Gene regulatory networks and pathways analysis

We downloaded clinical and RNA-seq data of PTC samples from TCGA website and used edgeR package in R to get differentially expressed genes using 234 $BRAF^{V600E}$-PTC and 255 $BRAF^{WT}$-PTC samples. Once, the differentially expressed genes were obtained, genes with p-value less than 0.05 were considered significant. WGCNA (weighted correlation network analysis) was used to build networks between differentially expressed genes obtained from $BRAF^{V600E}$-PTC versus $BRAF^{WT}$-PTC TCGA on the basis of co-expression information of genes. To understand RBMX and RBM10 focused molecular mechanisms and identify affected pathways, we extracted the interactome containing first neighbors of those molecules. The pathways analysis was performed in interactome using ingenuity pathways analysis system and pathways with p-value <0.05 were considered significantly affected. To understand interactions of cancer-associated genes (annotated in the NCBI data base) from chromosome 5 with RBMX and RBM10 genes we also generated non-thyroid specific interactive networks using GeneMANIA plugin in Cytoscape software (USA).

Quantitative multigene profiling mRNA expression analysis

Human cyclin B1 mRNA expression levels were validated by multi-gene transcriptional profiles which provides a quantitative view of the expression of many genes [11]. Cells were seeded at about 80% confluence in 6-well dishes and grown in high glucose DMEM supplemented with 0.2% FBS. Twenty-four hours after seeding, RNA isolation was performed by Quiagen columns (Quiagen, USA) following manufacturer protocols. Quantitative multi-gene profiling was performed by absolute quantification using real-time reverse transcriptase RT–PCR (RT-PCR) according to Shih et al. [11]. Primer sequences used for the validation of human cyclin B1 are the following: forward, AAATCAAGGACTTACAAAGCACATGA, and reverse, GCTGTGGTAGAGTGCTGATCTTAGC.

REFERENCES

1. Kurebayashi J, Tanaka K, Otsuki T, Moriya T, Kunisue H, Uno M, Sonoo H. All-trans-retinoic acid modulates expression levels of thyroglobulin and cytokines in a new human poorly differentiated papillary thyroid carcinoma cell line, KTC-1. J Clin Endocrinol Metab. 2000; 85:2889–96.
2. Nucera C, Nehs MA, Mekel M, Zhang X, Hodin R, Lawler J, Nose V, Parangi S. A novel orthotopic mouse model of human anaplastic thyroid carcinoma. Thyroid. 2009; 19:1077–84.
3. Nucera C, Porrello A, Antonello ZA, Mekel M, Nehs MA, Giordano TJ, Gerald D, Benjamin LE, Priolo C, Puxeddu E, Finn S, Jarzab B, Hodin RA, et al. B-Raf(V600E) and thrombospondin-1 promote thyroid cancer progression. Proc Natl Acad Sci USA. 2010; 107:10649–54.
4. Nehs MA, Nucera C, Nagarkatti SS, Sadow PM, Morales-Garcia D, Hodin RA, Parangi S. Late intervention with anti-BRAF(V600E) therapy induces tumor regression in an orthotopic mouse model of human anaplastic thyroid cancer. Endocrinology. 2012; 153:985–94.
5. Adamson B, Smogorzewska A, Sigoillot FD, King RW, Elledge SJ. A genome-wide homologous recombination screen identifies the RNA-binding protein RBMX as a component of the DNA-damage response. Nat Cell Biol. 2012; 14:318–28.
6. Li H, Durbin R. Fast and accurate long-read alignment with Burrows-Wheeler transform. Bioinformatics. 2010; 26:589–95.
7. McKenna A, Hanna M, Banks E, Sivachenko A, Cibulskis K, Kernytsky A, Garimella K, Altshuler D, Gabriel S, Daly M, DePristo MA. The Genome Analysis Toolkit: a MapReduce framework for analyzing next-generation DNA sequencing data. Genome Res. 2010; 20:1297–303.
8. DePristo MA, Banks E, Poplin R, Garimella KV, Maguire JR, Hartl C, Philippakis AA, del Angel G, Rivas MA, Hanna M, McKenna A, Fennell TJ, Kernytsky AM, et al. A framework for variation discovery and genotyping using next-generation DNA sequencing data. Nat Genet. 2011; 43:491–98.
9. Cibulskis K, Lawrence MS, Carter SL, Sivachenko A, Jaffe D, Sougnez C, Gabriel S, Meyerson M, Lander ES, Getz G. Sensitive detection of somatic point mutations in impure and heterogeneous cancer samples. Nat Biotechnol. 2013; 31:213–19.
10. McLaren W, Pritchard B, Rios D, Chen Y, Flicek P, Cunningham F. Deriving the consequences of genomic variants with the Ensembl API and SNP Effect Predictor. Bioinformatics. 2010; 26:2069–70.
11. Shih SC, Smith LE. Quantitative multi-gene transcriptional profiling using real-time PCR with a master template. Exp Mol Pathol. 2005; 79:14–22.

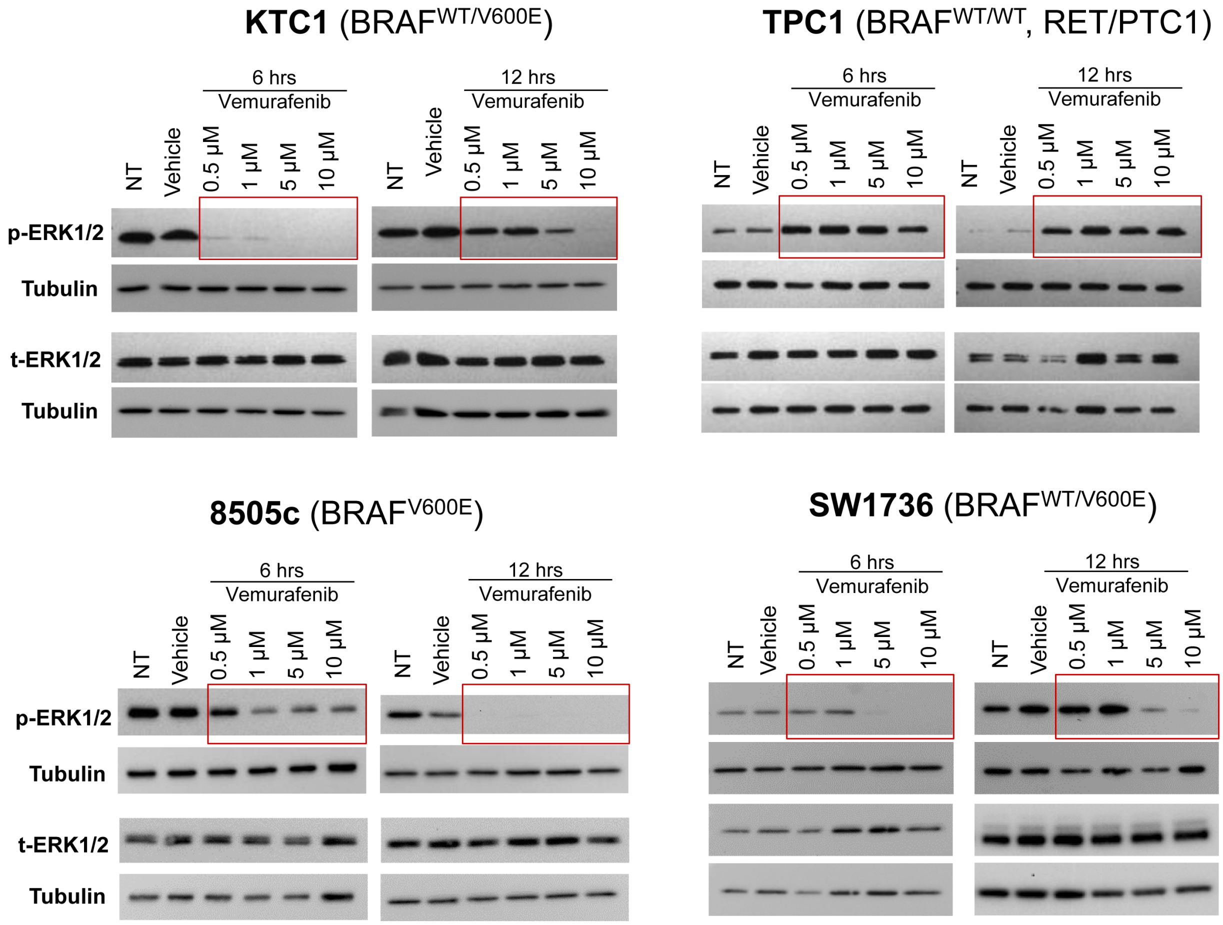

Supplementary Figure 1: Time course of phospho(p)-ERK1/2 protein expression levels in human thyroid carcinoma cells harboring BRAF^{V600E} mutation or with BRAF^{WT}. Western blot analysis of phospho(p)-ERK1/2 and total(t)-ERK1/2 protein expression levels in surviving KTC1 (PTC-derived), TPC1 (PTC-derived), 8505c (ATC-derived) and SW1736 (ATC-derived) cells treated with different doses (µM) of vemurafenib or vehicle (DMSO) at the indicated time points.

Naïve KTC1 cells

46,XY[12]

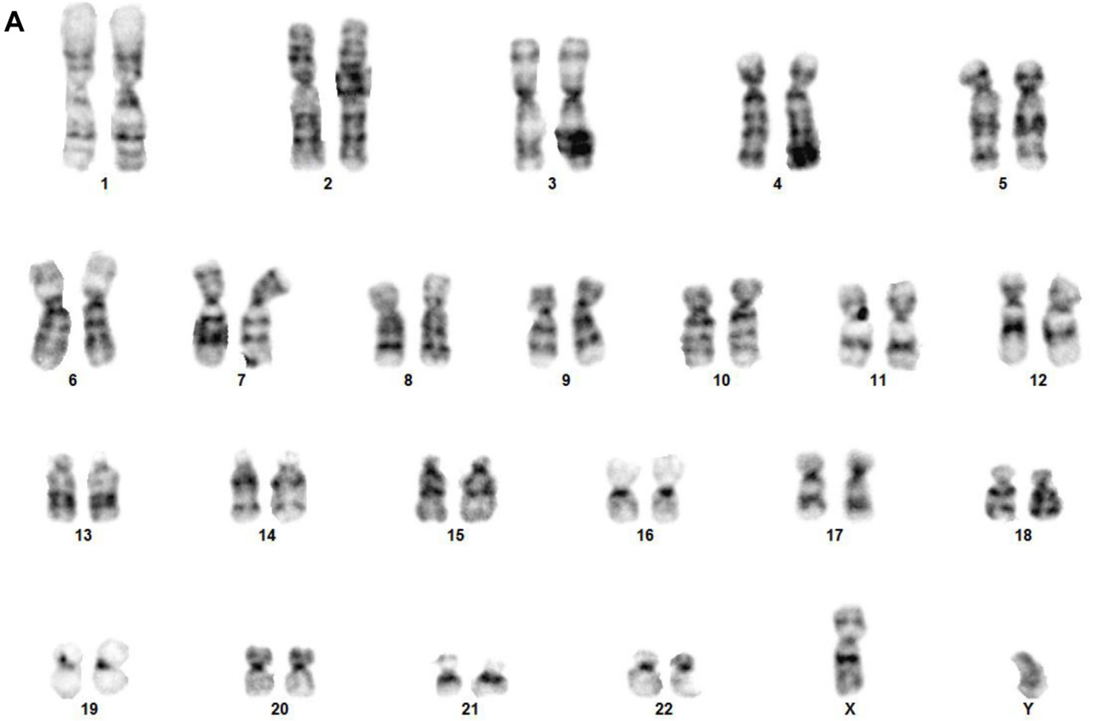

Naïve KTC1 cells

48,XY,+i(5)(p10)x2[8]

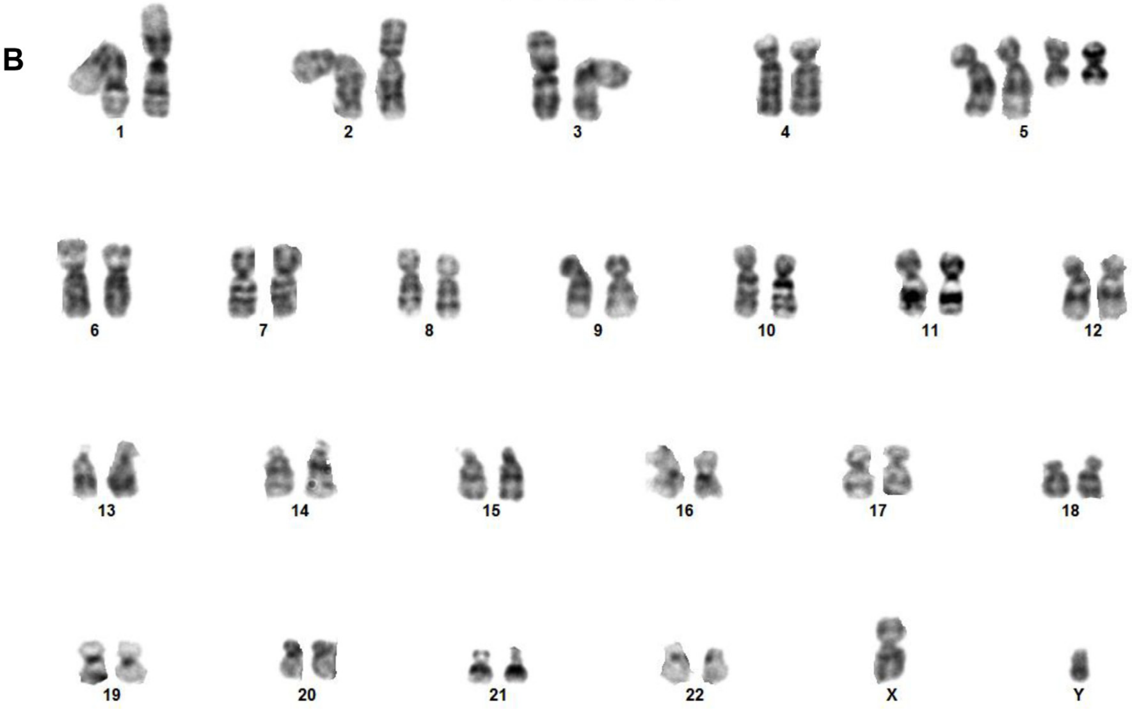

Res.1 KTC1 cells

46,XY[9]

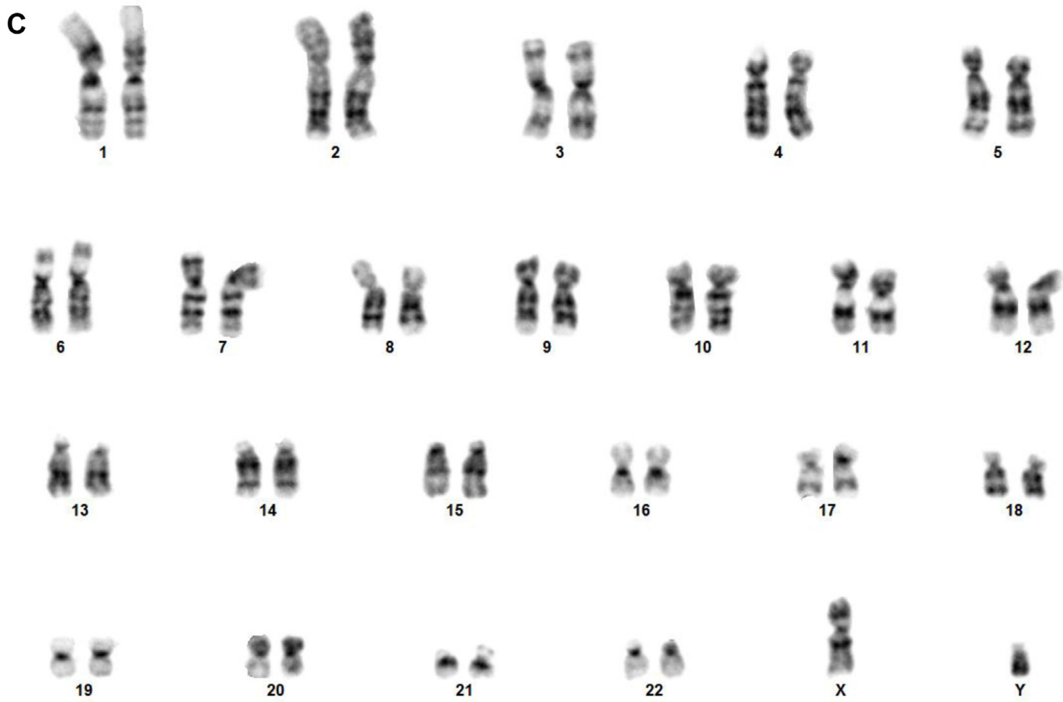

Res.1 KTC1 cells

47,XY,+5[9]

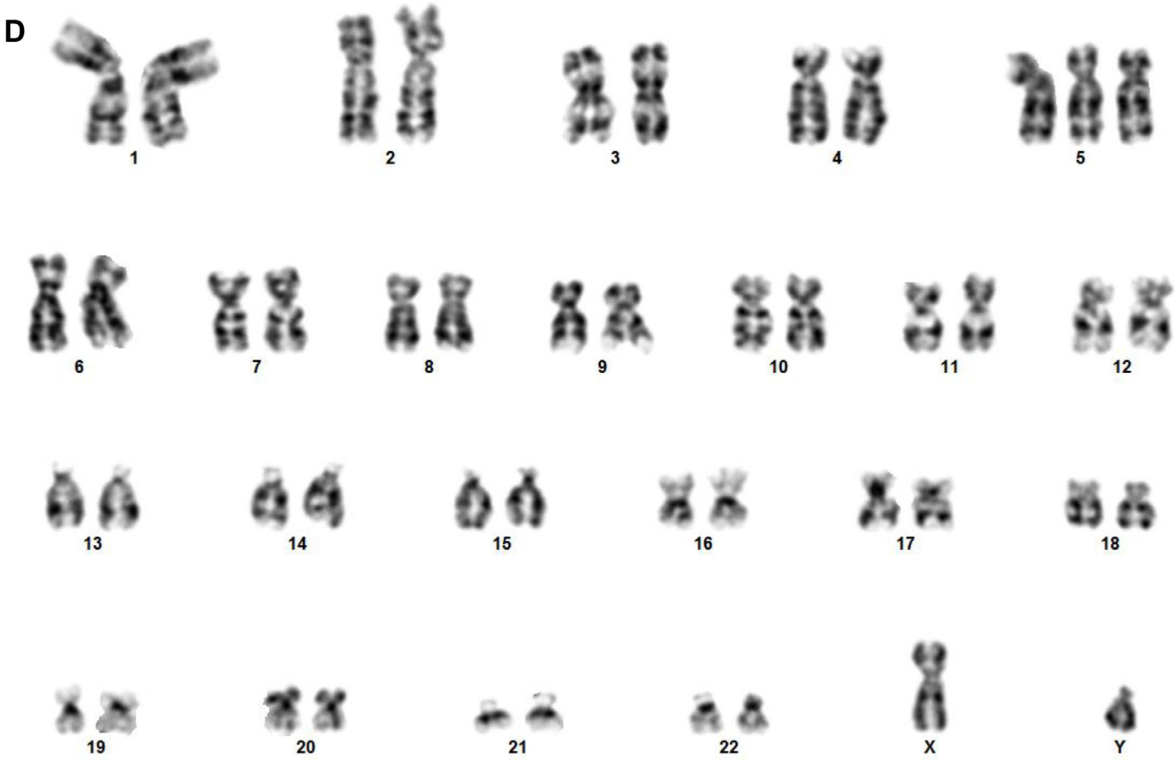

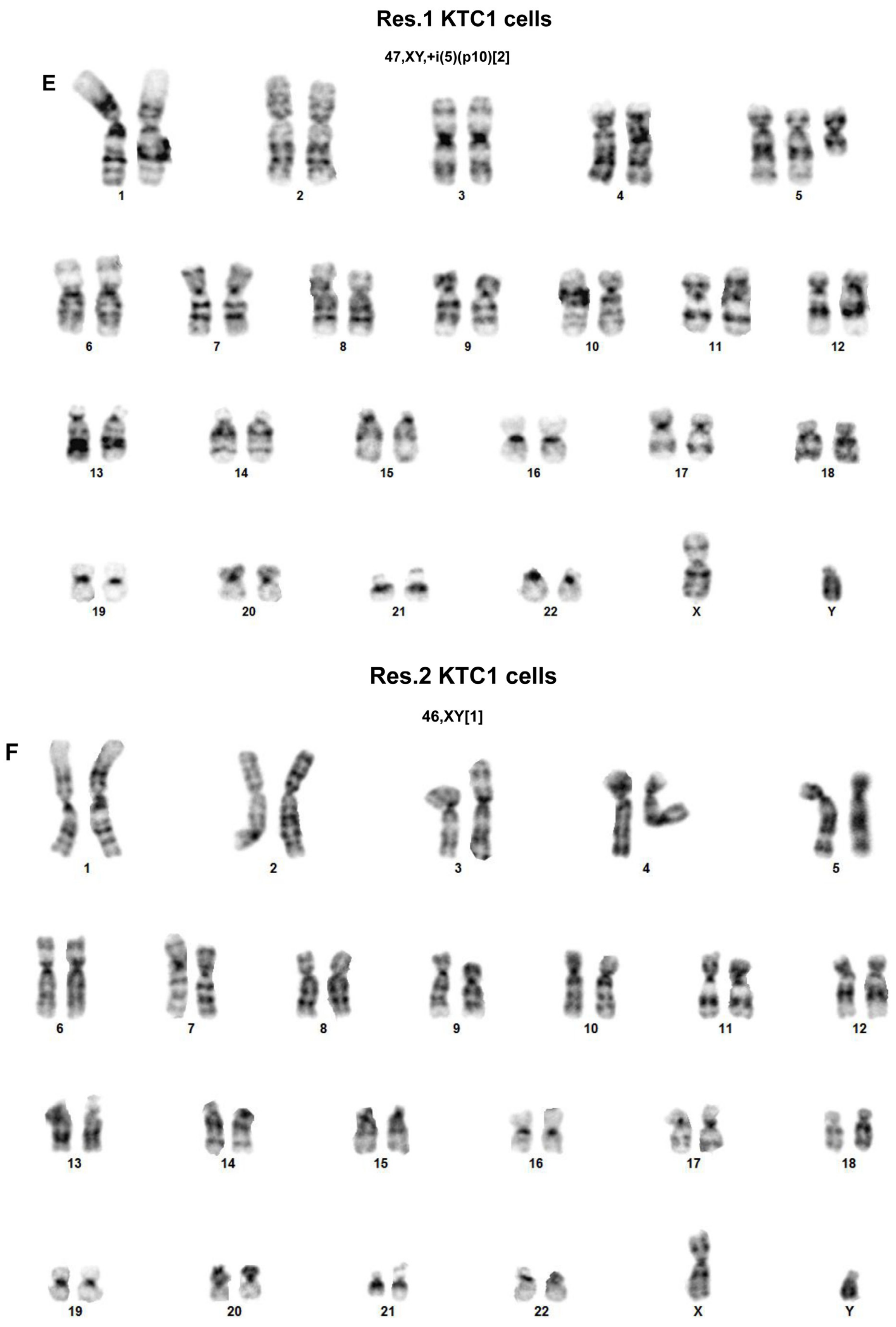
Res.1 KTC1 cells
47,XY,+i(5)(p10)[2]
E
1
2
3
4
5
6
7
8
9
10
11
12
13
14
15
16
17
18
19
20
21
22
X
Y
Res.2 KTC1 cells
46,XY[1]
F
1
2
3
4
5
6
7
8
9
10
11
12
13
14
15
16
17
18
19
20
21
22
X
Y

Res.2 KTC1 cells

47,XY,+i(5)(p10)[4]

G

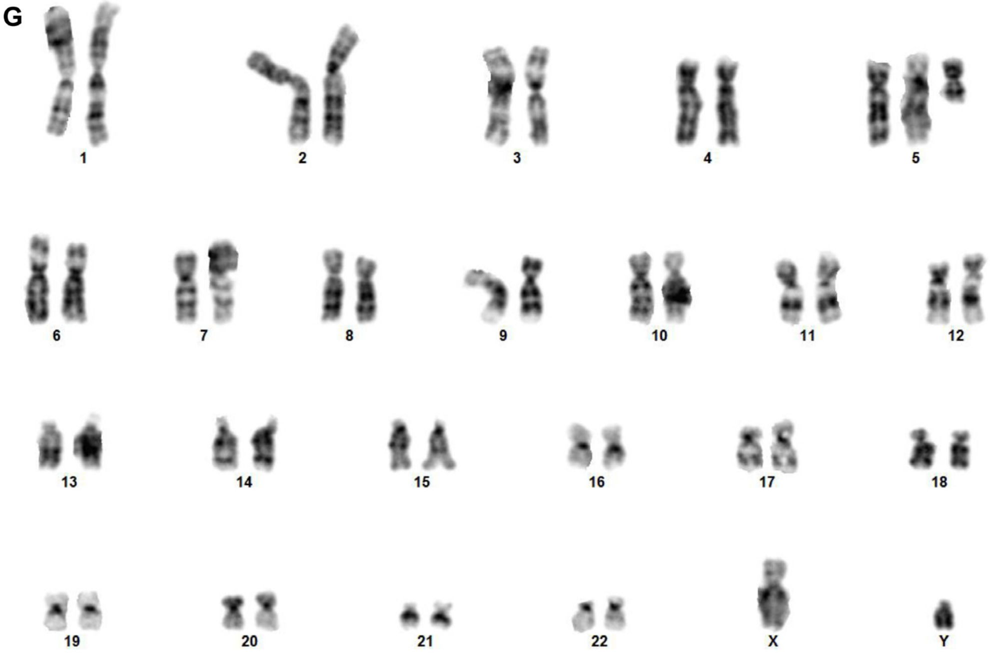

Res.2 KTC1 cells

48,XY,+Y,+i(5)(p10)[3]

H

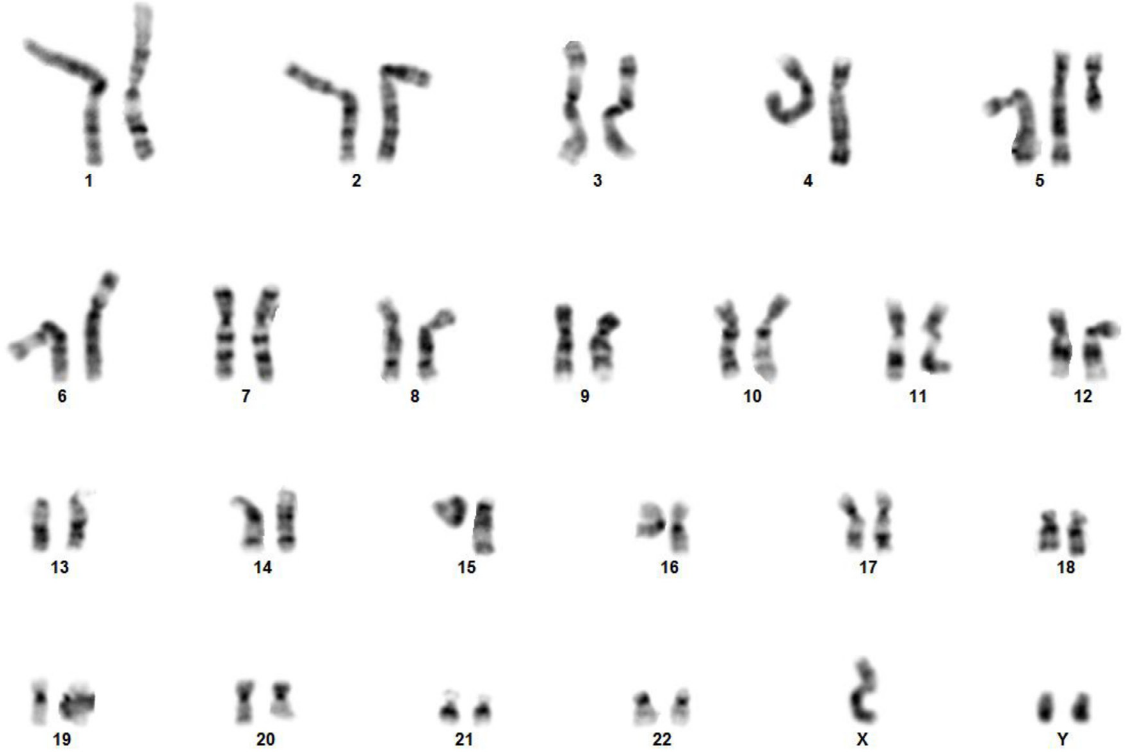

Supplementary Figure 2: Karyotype analysis in human thyroid carcinoma cells. Chromosomes banding analysis of KTC1^{Naive}, KTC1$^{Res.1}$, and KTC1$^{Res.2}$ cells. Karyotype categories show either diploid or aneuploid chromosomal patterns in KTC1 cells.

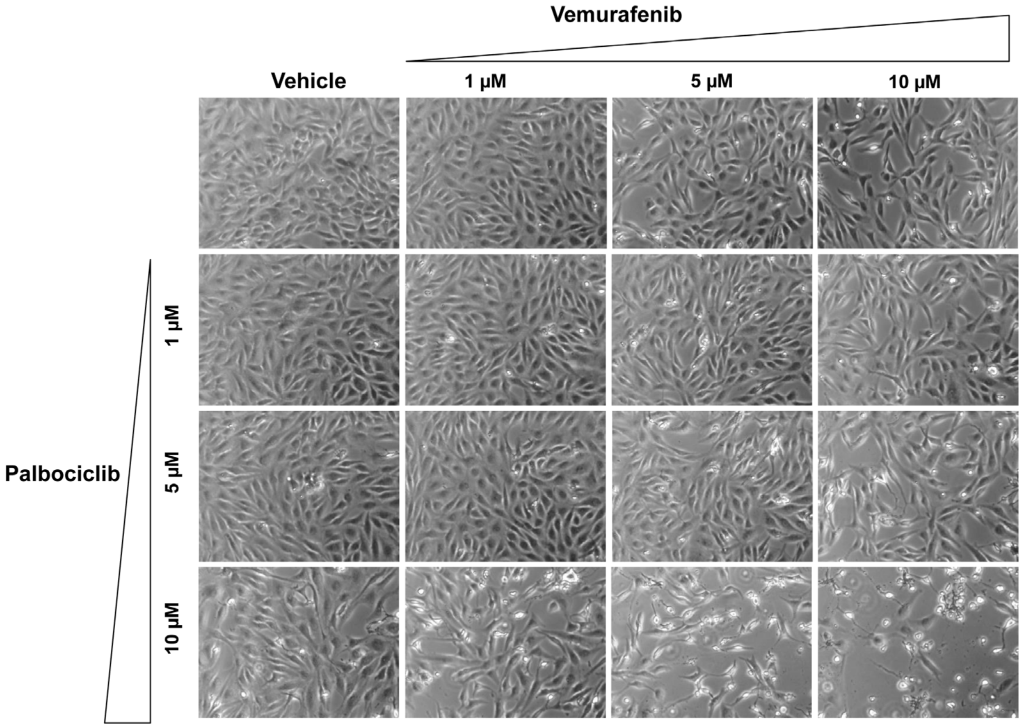

Supplementary Figure 3: Therapeutic efficacy of vemurafenib and palbociclib in KTC1 cells. Phase contrast images (Magnification: 10×) at 48 hours post-treatment of human heterozygous $BRAF^{WT/V600E}$-KTC1 cells treated with vehicle (DMSO); 1, 5 or 10 µM vemurafenib; 1, 5 or 10 µM palbociclib; or therapy with vemurafenib plus palbociclib combining all above doses.

Heterozygous $BRAF^{WT/V600E}$ SW1736 ATC cells

wild-type P16

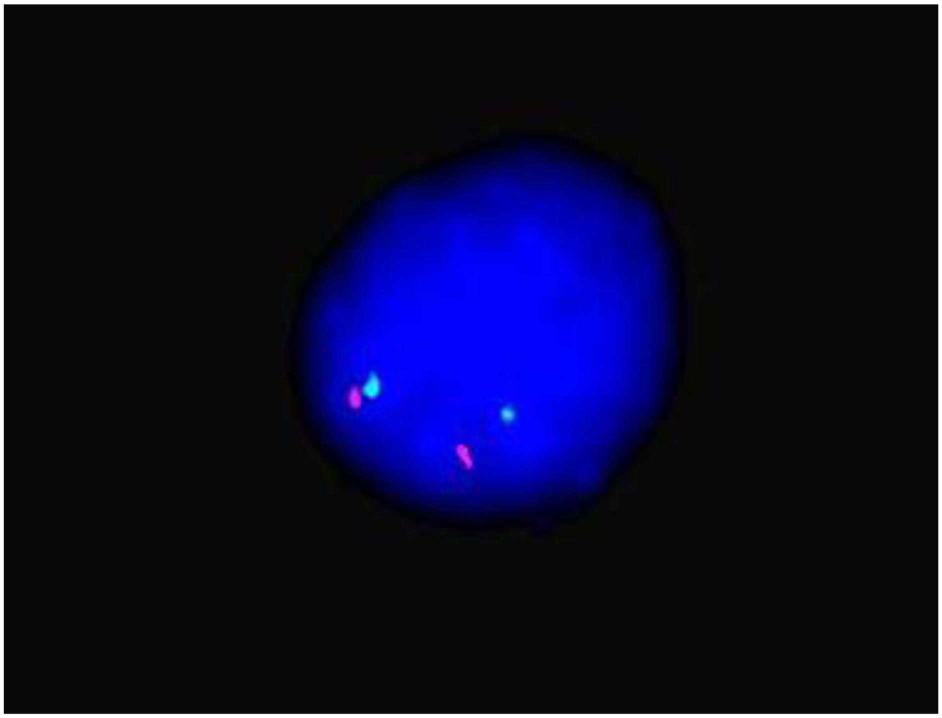

Supplementary Figure 4: Fluorescence *in situ* hybridization (FISH) analysis for the detection of P16 (CDKN2A) gene in the human anaplastic thyroid carcinoma-derived SW1736 cells. The red signal is the P16 gene (chromosome 9p21), and green signal is on the centromere of the chromosome 9. SW1736 cells show wild-type copy of P16.

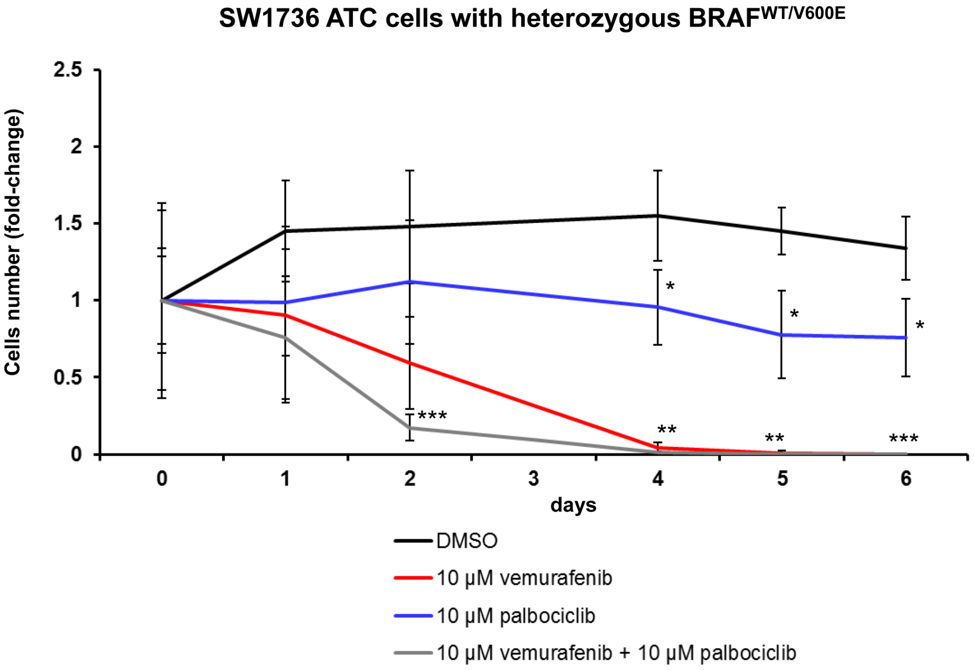

Supplementary Figure 5: Cell growth curve of human anaplastic thyroid carcinoma cells treated with vehicle, vemurafenib, palbociclib, or combined therapy. Fold changes calculation of cell growth of human heterozygous $BRAF^{WT/V600E}$-positive human anaplastic thyroid carcinoma (ATC)-derived SW1736 cells treated with 10 µM vemurafenib, 10 µM palbociclib, 10 µM vemurafenib plus 10 µM palbociclib or vehicle (DMSO). Statistical analysis is based on two-tailed Student's t-test comparison between vehicle-treated cells and drugs-treated cells. *= $p<0.05$; **= $p<0.01$; ***= $p<0.001$.

For Supplementary Tables see in Supplementary Files
